# Small heterodimer partner (SHP) aggravates ER stress in Parkinson’s disease-linked LRRK2 mutant astrocyte by regulating XBP1 SUMOylation

**DOI:** 10.1186/s12929-021-00747-1

**Published:** 2021-07-07

**Authors:** Jee Hoon Lee, Ji-hye Han, Eun-hye Joe, Ilo Jou

**Affiliations:** 1 Department of Pharmacology, Ajou University School of Medicine, Suwon, 442-721 South Korea; 2 Inflamm-Aging Translational Research Center, Ajou University School of Medicine, Suwon, South Korea

**Keywords:** Parkinson’s diseases, LRRK2, ER stress, Astrocytes, XBP1, SUMOylation, SHP, PIAS1

## Abstract

**Background:**

Endoplasmic reticulum (ER) stress is a common feature of Parkinson’s disease (PD), and several PD-related genes are responsible for ER dysfunction. Recent studies suggested LRRK2-G2019S, a pathogenic mutation in the PD-associated gene *LRRK2*, cause ER dysfunction, and could thereby contribute to the development of PD. It remains unclear, however, how mutant LRRK2 influence ER stress to control cellular outcome. In this study, we identified the mechanism by which LRRK2-G2019S accelerates ER stress and cell death in astrocytes.

**Methods:**

To investigate changes in ER stress response genes, we treated LRRK2-wild type and LRRK2-G2019S astrocytes with tunicamycin, an ER stress-inducing agent, and performed gene expression profiling with microarrays. The XBP1 SUMOylation and PIAS1 ubiquitination were performed using immunoprecipitation assay. The effect of astrocyte to neuronal survival were assessed by astrocytes-neuron coculture and slice culture systems. To provide in vivo proof-of-concept of our approach, we measured ER stress response in mouse brain.

**Results:**

Microarray gene expression profiling revealed that LRRK2-G2019S decreased signaling through XBP1, a key transcription factor of the ER stress response, while increasing the apoptotic ER stress response typified by PERK signaling. In LRRK2-G2019S astrocytes, the transcriptional activity of XBP1 was decreased by PIAS1-mediated SUMOylation. Intriguingly, LRRK2-GS stabilized PIAS1 by increasing the level of small heterodimer partner (SHP), a negative regulator of PIAS1 degradation, thereby promoting XBP1 SUMOylation. When SHP was depleted, XBP1 SUMOylation and cell death were reduced. In addition, we identified agents that can disrupt SHP-mediated XBP1 SUMOylation and may therefore have therapeutic activity in PD caused by the LRRK2-G2019S mutation.

**Conclusion:**

Our findings reveal a novel regulatory mechanism involving XBP1 in LRRK2-G2019S mutant astrocytes, and highlight the importance of the SHP/PIAS1/XBP1 axis in PD models. These findings provide important insight into the basis of the correlation between mutant LRRK2 and pathophysiological ER stress in PD, and suggest a plausible model that explains this connection.

**Supplementary Information:**

The online version contains supplementary material available at 10.1186/s12929-021-00747-1.

## Background

Parkinson’s disease (PD), the second most common age-related neurodegenerative disease, is associated with the appearance of several motor symptoms, including rigidity, resting tremor, bradykinesia, and postural instability [9]. The pathological hallmarks underlying the clinical phenotypes are triggered by the loss of dopaminergic neurons in the substantia nigra pars compacta (SNpc) [8]. Recent studies suggest that non-neuronal cells such as astrocytes can contribute to neurodegeneration through various pathways [10, 49]. This suggests that astrocytes play critical roles in neuronal dysfunction, and that the interplay between astrocytes and neurons could provide insights into the mechanisms underlying neuronal dysfunction and death in PD [4]. Despite this, there have been few studies into the function in PD.

Studies of the human PD brain have revealed several perturbations in cellular homeostasis associated with PD, including alterations in mitophagy, calcium regulation, energy metabolism, redox balance, and endoplasmic reticulum (ER) stress [32]. The ER provides the environment for the folding and post-translational modification of all secreted proteins in eukaryotic cells. When demand exceeds capacity, unfolded proteins accumulate in the ER lumen, causing ER stress. This stress, in turn, activates three ER-transmembrane unfolded protein response (UPR) sensors, IRE1, PERK and ATF6, which transduce signals to decrease protein translational load while increasing ER folding capacity. One of the sensors, IRE1, activates the transcription factor XBP1 to induce genes that facilitate protein folding and removal of unfolded proteins by ER-associated degradation (ERAD), including some that encode ER chaperones [14, 42, 45, 54]. Active IRE1 can also cleave ER-localized mRNAs in a process known as regulated IRE1-dependent decay (RIDD), which further decreases ER translational load and helps to restore homeostasis [16]. However, if stress persists, the cell undergoes apoptosis. Another UPR sensor, PERK, drives apoptotic cell death through ATF4-dependent induction of the transcription factor CHOP, which induces expression of caspases [38]. PERK and IRE1 signaling are critical for cell fate determination during ER stress. Under irremediable ER stress, PERK apoptotic function predominates, while signaling mediated by IRE1/XBP1 is attenuated by an unknown mechanism. Although there are few studies the UPR activation in astrocytes can modulate pathogenesis of neurodegenerative disease [24, 48, 55], these UPR signaling activation pattern in astrocytes during diseases and how this impacts on pathogenesis remain unclear.

Recent studies suggest that several PD-related genes are responsible for ER dysfunction [30, 31]. A major genetic form of PD is caused by a mutation in LRRK2 [33, 61]. The Gly2019Ser (G2019S) mutation is the most common LRRK2 mutation and, as such, one of the most common causes of PD [28]. Studies in *Caenorhabditis elegans* have demonstrated that LRRK2 is critical for preventing ER stress and dopaminergic neuronal death [44, 59]. In addition, we previously suggested that the LRRK2-G2019S mutation impairs ER Ca^2+^ homeostasis, which determines cell survival, and could thereby contribute to the development of PD [24]. Despite these advances in our understanding, the contribution of ER stress to the pathogenic manifestations of mutant LRRK2 remain largely unknown.

In this study, we identified the mechanism by which LRRK2-G2019S accelerates ER stress and cell death in astrocytes. Gene expression profiling of LRRK2-G2019S astrocytes revealed that expression of XBP1 target genes decreased, whereas expression of PERK-induced apoptotic genes increased. LRRK2-G2019S negatively regulated XBP1 transcriptional activity by promoting PIAS1-mediated SUMOylation of XBP1, but did not affect phosphorylation and oligomerization of IRE1. LRRK2-G2019S increased expression of small heterodimer partner (SHP), an orphan nuclear receptor, thereby stabilizing the PIAS1 protein and promoting XBP1 SUMOylation. When SHP was depleted, XBP1 SUMOylation and cell death were reduced. In addition, we identified agents that can disrupt SHP-mediated XBP1 SUMOylation and may therefore have therapeutic activity in PD caused by the LRRK2-GS mutation.

## Materials and methods

### Animals

G2019S-*LRRK2*-Tg and wild type-*LRRK2*-Tg FVB mice were purchased from Jackson Laboratory (Bar Harbor, ME, USA). Wild type-*LRRK2*-Tg and G2019S-*LRRK2*-Tg heterozygous mice were prepared by crossing non-Tg mice with each type of *LRRK2*-Tg mice, respectively. Genotyping was carried according to the vendor’s instructions. All animal procedures were approved by the Ajou University Institutional Animal Experimentation Committee (AMC-119).

### Stereotaxic injection and tissue preparation

The wild type-*LRRK2*-Tg and G2019S-*LRRK2*-Tg (8-week-old) were anesthetized by i.p. injection of 2.5% avertin and positioned in a stereotaxic apparatus (David Kopf Instruments, USA). Tunicamycin (1 μg/μl) in 1 μl saline was unilaterally administered into the cortex (AP, + 1.0 mm; ML, + 1.6 mm; DV, − 1.2 mm from bregma), according to the atlas of Paxinos and Watson [35]. All animals were injected using a Hamilton syringe equipped with a 33-gauge blunt needle attached to a syringe pump (KD Scientific, USA). Tunicamycin was infused at a rate of 0.4 μl/min. After injection, the needle was held in place for an additional 5 min before removal. DMSO-injected cortexes were used as controls. We collected data from 4 to 5 animals for each insult.

For immunohistochemistry, mice were anesthetized and transcardially perfused with saline solution containing 0.9% sodium chloride, 0.5% sodium nitrate and heparin (10 U/ml), followed by 10% formalin in 0.1 M phosphate buffer (pH 7.2) for tissue fixation. Brains were separated and post-fixed overnight at 4 °C in 4% paraformaldehyde. Fixed brains were stored at 4 °C in a 30% sucrose solution until they sank. The separate series of 12-μm coronal brain sections were obtained using a sliding microtome (Microm, Germany) and stored in antifreeze stock solution (phosphate buffer pH 7.2 containing 30% glycerol, 30% ethylene glycol) at 4 °C before use.

### Immunofluorescence

Brain sections were blocked in 1% bovine serum albumen (BSA) with 0.2% Triton X-100, before being incubated overnight at 4 °C with mouse anti-CHOP diluted in 1% BSA with 0.2% Triton X-100 in PBS. Sections were washed in PBS before being incubated with secondary antibody. Sections were washed in PBS, then mounted with Vectashield medium containing DAPI (Vector Laboratories, Burlingame, CA, USA).

Astrocytes and neurons isolated from LRRK2-WT and LRRK2-GS mice were treated with tunicamycin (0.2 μg/ml) and then fixed with 4% paraformaldehyde and then permeabilized with 0.25% triton for 10 min. Cells were washed three times in PBS and blocked with 1% BSA for 1 h. Cells were incubated with primary antibodies overnight at 4 °C. Washed cells were incubated with fluorescent secondary antibody (Invitrogen) for 1 h at room temperature. Cover slips were mounted with Vectashield medium containing DAPI and viewed on confocal microscope confocal microscope (TCS, DMi8; Leica, Germany).

### Cell culture

Primary astrocytes were cultured from the cerebral cortices of 1-d-old wild type-*LRRK2*-Tg and G2019S-*LRRK2*-Tg heterozygous mice. Briefly, cortices were triturated into single cells in Dulbecco’s modified Eagle’s medium (DMEM; Sigma-Aldrich, St. Louis, MO, USA) containing 10% (v/v) fetal bovine serum (FBS; Hyclone, South Logan, UT, USA), plated into 75 cm^2^ T-flasks, and incubated for 2 week. Following removal of microglia, primary astrocytes were enzymatically dissociated with trypsin (Sigma) for 5 min at 37 °C in a humidified 5% CO_2_, 95% air chamber. Trypsinization was quenched by adding astrocyte culture medium and centrifuged (~ 200*g*) for 5 min. Microglia and meningeal cells were depleted by incubating astrocytes with serum-free DMEM for 2 d before use. The cell populations obtained consisted of more than 95% authentic astrocytes, as determined using the astrocyte marker GFAP (glial fibrillary acidic protein) immunofluorescence.

Primary neurons were cultured from embryonic mouse cortices (E17). Briefly, cortices were dissected in Hank’s Buffered Salt Solution (HBSS; Gibco, Carlsbad, CA, USA) supplemented with HEPES (10 mM, pH 7.4). Tissues were gently triturate with the fire-polished Pasteur pipette dissociated cells were plated in poly-d-lysine (1 mg/ml)-coated 6-well plate (3 × 10^5^ cells/well) or 12-mm cover glasses (1 × 10^4^ cells) in Neurobasal medium containing B27 (2%), sodium pyruvate (1%), penicillin/streptomycin (1%), and GlutaMax (1%) (all supplements were from Gibco). Cells were incubated for 10 d and then challenged with tunicamycin for 3–48 h. For co-culture with astrocytes, cortical neurons were seeded on LRRK2-WT or LRRK2-GS astrocyte monolayers for 10 d, then treated with tunicamycin (Sigma) for 24–48 h and assayed by immunocytochemistry.

### Organotypic brain slice culture

Organotypic brain slice cultures were prepared from postnatal day 7 LRRK2-WT and LRRK2-GS mice. Mice were anesthetized, and cortices were dissected and coronally sectioned (250 μm) using a McIlwain tissue chopper (Mickle Laboratory Engineering, UK). Slices were mounted on Millicell cell culture inserts (0.4 μm pore size, 30 mm diameter; Millipore, Burlington, MA, USA). Culture medium (50% minimal essential medium [MEM] containing 25% HBSS, 25% heat-inactivated horse serum, 0.5% glucose, 1 mM l-glutamine) was changed every 2–3 d. Slices were treated with tunicamycin (0.5 μg/ml) after 7 d in culture.

### Gene expression profiling analysis

The astrocytes from LRRK2-WT and LRRK2-GS mice were stimulated with tunicamycin (0.2 μg/ml) and total RNA was extracted using RNAiso Plus (Takara, Japan) and purified on RNeasy columns (Qiagen, Germany). Complementary DNA (cDNA) was hybridized to each GeneChip^®^ Mouse 2.0 ST Array (Affymetrix, Santa Clara, CA, USA). Array data export processing and analysis were performed using Affymetrix GeneChip Command Consol^®^ software. Statistical significance of the expression data was determined using fold change and independent t tests, in which the null hypothesis was that there would be no difference among groups. The false discovery rate (FDR) was controlled by adjusting the P value using the Benjamini–Hochberg algorithm. The genes showing significant changes (> 1.5-fold or < 1.5-fold; FDR-adjusted P ≤ 0.05) was listed. The list of significant probes was determined by gene enrichment and functional annotation analysis using Gene Ontology software (http://geneontology.org/). Sequencing and statistical analysis were conducted by Macrogen Inc. (Korea). Microarray data have been deposited in the National Center for Biotechnology Information (NCBI) Gene Expression Omnibus (GEO) under Accession number GSE139579.

### Plasmid constructs

Plasmid DNA for Myc-tagged mouse SHP and Myc-tagged mouse XBP1s was purchased from Origene (Rockville, MD, USA). To construct the plasmid encoding the luciferase gene driven by XBP1-binding sites, a synthetic oligonucleotide containing four XBP1-binding sites, 5ʹ- aagctagccgcgTGGA*GCCACGT*TACATGGA*GCCACGT*TACATGGA*GCCACGT*TACATGGA*GCCACGT*TACAaagctttt-3ʹ (italicized sequences represent the XBP1-binding sites), was digested with NheI/HindIII and the ligated into the pGL3 vector. Site-directed mutagenesis to generate XBP1s mutants was performed using Quick Change Site-Directed Mutagenesis Kit (Stratagene, La Jolla, CA) according to the manufacturer’s instructions. The correct introduction of the mutants was confirmed by DNA sequencing. All primers used in mutagenesis are listed Additional file 1: Table S2.

### Luciferase reporter assay

Astrocytes isolated from LRRK2-WT or LRRK2-GS mice were transfected with expression plasmids by Lipofectamine 2000 (Invitrogen, Carlsbad, CA, USA). The cells were incubated for 24 h before luciferase was assayed using Dual-Luciferase Reporter Assay System (Promega, Madison, WI, USA). Renilla luciferase activity was used as a control.

### TUNEL assay

TUNEL assays were carried out using a commercial kit according to the manufacturer’s instructions (Invitrogen). Briefly, cells and proteinase K-treated organotypic slices were rinsed with phosphate-buffered saline (PBS), and then incubated in 1× equilibration buffer for 10 min. Thereafter, samples were incubated with terminal deoxynucleotidyl transferase (TdT) for 1 h at 37 °C, blocked with stop/wash buffer, and incubated with peroxidase antibody for 30 min at room temperature. The images were taken from layer 1 of the cortex in brain slices (n = 5 slices from 3 mouse) and the percentage of TUNEL-positive cells was determined in at least 10 optical fields.

### Live and dead assay

Live and dead assays were carried out using kinetic apoptosis kit according to the manufacturer’s instructions (Abcam, UK). Briefly, cells were treated with propidium iodide (PI) and Annexin XII based pSIVA (Polarity Sensitive Indicator of Viability & Apoptosis) probe for 10 min. Thereafter, fluorescence density was analyzed by confocal microscope and Cytation 5 Cell Imaging Multimode Reader (BioTek, Winooski, VT, USA). Use the green fluorescence filter set for pSIVA (excitation maximum 488 nm and emission maximum 530 nm) and a red fluorescence filter set for PI.

### Proximity Ligation Assay (PLA)

Fixed cells were stained with the indicated rabbit and mouse antibodies. Duolink-PLA (Olink Bioscience, Sweden) procedures were performed according to the manufacturer’s instructions. Each discrete red spot represents a protein–protein complex (radius < 40 nm). Micrographs from ten independent areas per group were analyzed.

### Chromatin immunoprecipitation (ChIP) assay

ChIP analysis was performed on astrocytes from LRRK2-WT and LRRK2-GS mouse using the Pierce™ Magnetic ChIP Kit (Thermo Fisher, Waltham, MA, USA) according to the manufacturer’s instructions. Briefly, cells were crosslinked with 1% (v/v) formaldehyde and then adducts were digested using micrococcal nuclease to get DNA fragments of 500–1000 bp. Supernatants were collected and immunoprecipitated with anti-XBP1 antibody overnight at 4 °C. The protein-bound, immunoprecipitated DNA was recovered, and qRT-PCR was performed using the primer pairs (Additional file 1: Table S2). Normalization was performed to input using the formula

$${\text{Input}}\% = 100/2^{{\left( {{\text{Ct}}\left[ {{\text{ChIP}}} \right] - {\text{Ct}}\left[ {{\text{Input}}} \right] - Log2\left( {{\text{Input Dilution Factor}}} \right)} \right)}} .$$

### Synthesis and transfection of siRNA

siRNA duplex oligonucleotides were chemically synthesized by Bioneer (Korea) and Santa Cruz (Santa Cruz, Dallas, TX, USA). Details of the siRNA sequences are described in Additional file 1: Table S3. Confluent astrocytes were transfected with siRNA oligonucleotides (50 μM) using Lipofectamine RNAiMax reagent (Invitrogen) according to the manufacturer’s instructions. All assays were performed at least 48 h after RNAi transfection.

### Immunoprecipitation of endogenous SUMOylated XBP1s

Astrocytes were co-transfected with siRNA targeting the 3ʹ-UTR region of XBP1s and Myc-tagged XBP1s-K276R or XBP1s-K298R. After 24 h, cells were treated with tunicamycin (0.2 μg/ml) for 24 h and then lysates were prepared using modified RIPA buffer containing 10 mM *N*-ethylmaleimide (NEM), a SUMO protease inhibitor. Lysates were immunoprecipitated using an XBP1s-conjugated magnetic beads (Invitrogen) and subjected to SDS-PAGE and Western blotting analysis. The SUMOylation of endogenous XBP1s was examined using an anti-XBP1s or anti-SUMO antibody.

### Western blotting

Cells were lysed with RIPA buffer (50 mM Tris–HCl pH 7.5, 150 mM NaCl, 1% Triton X-100, 1% sodium deoxycholate, 0.1% SDS, 2 mM EDTA pH 8.0) supplemented with a protease inhibitor cocktail (GenDEPOT, Barker, TX, USA) at 4 °C for 30 min. Samples were separated by SDS-PAGE and transferred to nitrocellulose membranes. Membranes were incubated with primary antibodies (Additional file 1: Table S4) and horseradish peroxidase (HRP)-conjugated secondary antibodies, and immunoreactive proteins were visualized using an enhanced chemiluminescence system (Ab Frontier, Korea).

### Real time quantitative reverse transcription-polymerase chain reaction (RT-qPCR) analysis

Total RNA was isolated using RNAiso Plus (TaKaRa, Japan), and cDNA was synthesized using avian myeloblastosis virus reverse transcriptase (New England Biolabs, Ipswich, MA, USA) and oligo (dT) primers (Promega), according to the manufacturers’ instructions. For qPCR, amplification reactions were performed using a Thermal Cycler Dice Real-Time System (TaKaRa) with SYBR Premix Ex Taq master mix (TaKaRa) according to the manufacturer’s instructions. The primers used for qPCR (Bioneer) are described in Additional file 1: Table S2.

### Statistical analysis

Prism 7.0 (GraphPad Software) was used for the statistical analyses. The significance of differences between groups was determined using unpaired two-tailed Student’s *t*-test or Kruskal–Wallis test followed by Dunn’s multiple comparisons test. A *p-*value of 0.05 was considered significant. Values are presented as means ± standard deviation (SD).

## Results

### XBP1 signaling is reduced in LRRK2-GS astrocytes

**Fig. 1:**
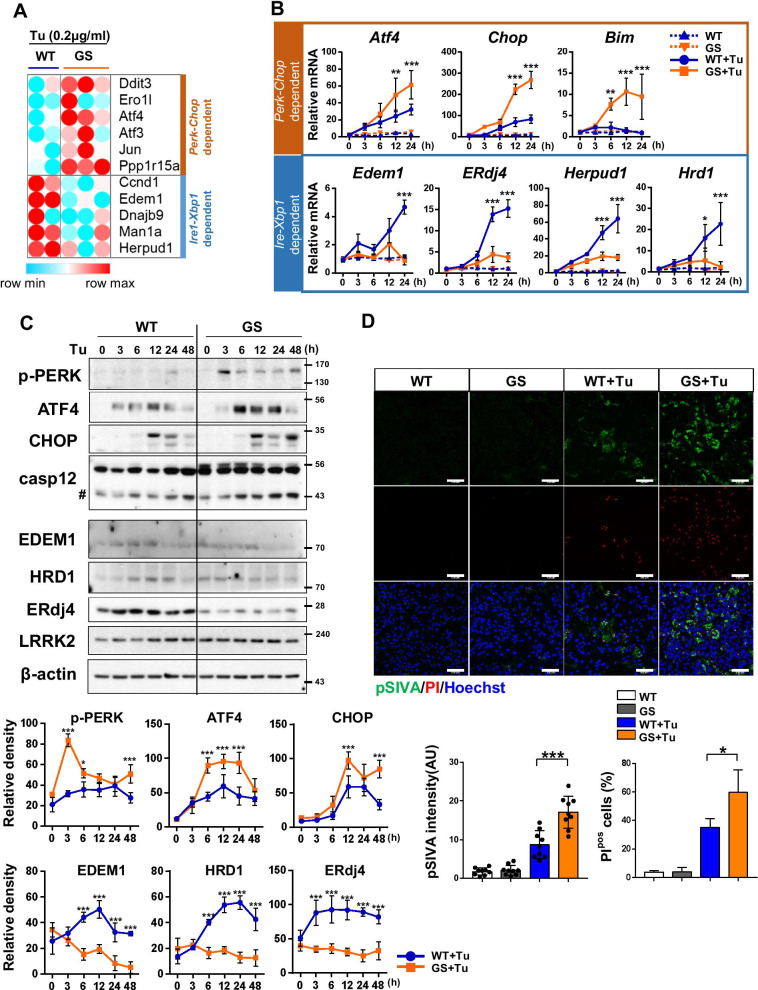
XBP1 signaling is attenuated in LRRK2-GS astrocytes. **A** DNA microarray analysis of LRRK2-WT (W) and -GS (G) astrocytes treated with 0.1 μg/ml tunicamycin (Tu) for 24 h. Heatmap showing ER stress-related genes differentially regulated in these samples (see Additional file 1: Fig. S1A, Table S1). **B**, **C** Astrocytes isolated from LRRK2-WT (WT) and -GS (GS) mice were treated with tunicamycin for indicated time points. Expression levels of the indicated mRNAs (**B**) and proteins (**C**) were analyzed by real-time qPCR and western blotting (WB), respectively. Crosshatching denotes cleaved caspase-12. Western blot band intensities were quantified using an Image J program (**C**, lower graph). Data are means ± SD of three independent experiments; **p < 0.01, ***p < 0.001. **D** LRRK2-WT and -GS astrocytes were treated with tunicamycin for 72 h. pSIVA + PI was added directly to the culture medium and cells were imaged (37 ℃, 5% CO_2_). Representative images and summary data showing apoptotic cells. Four fields of view per group of three independent experiments (n = 12). Scale bar, 100 μm. Data are means ± SD (*p < 0.05, **p < 0.01)

To investigate changes in UPR genes, LRRK2-wild type (LRRK2-WT) and LRRK2-G2019S (LRRK2-GS) astrocytes were treated with ER stressor tunicamycin (Tu) for 24 h and performed gene expression profiling with microarrays. We confirmed that LRRK2 is expressed in astrocytes at the same level in WT and GS animals and the high purity of astrocytes was confirmed by examining well described markers of CNS cell types (Additional file 1: Fig. S1A). Analysis of filtered microarrays revealed that expression of 358 genes was altered in LRRK2-GS cells. The differentially regulated genes were subjected to Gene Ontology (GO) analysis. Bioinformatics resources revealed enrichment of several functional categories in the list of genes, including protein synthesis, transmembrane transport system, and, as expected, the UPR (Additional file 1: Table S1). Intriguingly, comparison between the expression ratios for the UPR genes in the LRRK2-WT versus LRRK2-GS astrocytes revealed that expression of target genes from PERK/CHOP signaling was elevated, whereas expression of IRE1/XBP1 target genes was reduced, in LRRK2-GS astrocytes (Fig. 1A; Additional file 1: Fig. S1B). The microarray results were validated by qRT-PCR and western blotting for representative targets of PERK (*Atf4*, *Chop*, and *Bim*) and IRE1 (*Edem1*, *ERdj4*, *Herpud1*, and *Hrd1*) at each temporal points (Fig. 1B, C). Thus, PERK activity persisted, whereas IRE1 signaling was attenuated, in LRRK2-GS astrocytes. Because IRE1/XBP1 signaling positively regulates cell survival by adjusting protein-folding capacity, we investigated whether astrocyte death occurred in XBP1-knockdown cells. After introducing a siRNA targeting XBP1, we confirmed a reduction in expression of XBP1 target genes and protein, and an increase in expression of cell death markers (e.g., activation of caspase-12 and -3) and the proportion of TUNEL-positive cells. Overexpression of XBP1s, the active form of XBP1, in XBP1-knockdown cells abolished the increase in cell death, suggesting that IRE/XBP1 signaling is indispensable for astrocyte survival (Additional file 1: Fig. S1C, D). Based on our observation that IRE1/XBP1 signaling was reduced in LRRK2-GS astrocytes, we speculated that the rate of cell death would be elevated. To investigate the occurrence of apoptotic processes, we performed kinetic apoptosis assay using the pSIVA-IANBD polarity sensitive probe, which binds to phosphatidylserine exposed on the surface of apoptotic cells, and propidium iodide (PI), which selectively stains the nuclei of damaged cells. The data confirmed that the pSIVA/PI positive apoptotic cells was higher in LRRK2-GS vs. LRRK2-WT astrocytes (Fig. 1D). Taken together, these results confirm that IRE1/XBP1 signaling was reduced, and ER stress-mediated cell death was significantly elevated, in LRRK2-GS astrocytes.

### Transcriptional activity of XBP1 is reduced in LRRK2-GS astrocytes

**Fig. 2:**
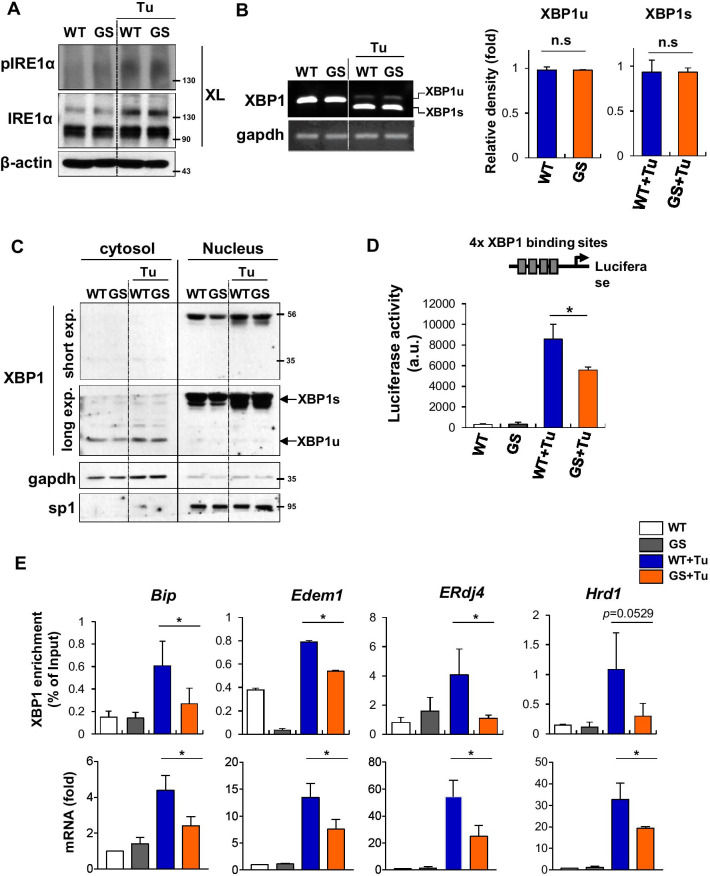
Transcriptional activity of XBP1 is reduced in LRRK2-GS astrocytes. **A** LRRK2-WT and -GS astrocytes were treated with tunicamycin. Cell lysates were subjected to chemical crosslinking (XL) with disuccinimidyl suberate (DSS), to stabilize IRE1 oligomers, and were analyzed by WB. **B** XBP1 mRNA splicing was analyzed by RT-PCR using total RNA isolated from LRRK2-WT and -GS astrocytes. The ratio of XBP1 was quantified (right panel). **C** Western blot analysis of XBP1 in the cytosol and nuclear fractions of LRRK2-WT and -GS astrocytes. *Gapdh* and *Sp1* were used as cytosolic and nuclear loading controls, respectively. **D** Luciferase reporter assay in LRRK2-WT and -GS astrocytes. Luciferase gene expression was driven by a synthetic promoter consisting of four tandem XBP1-binding sites. Data are means ± SD of three independent experiments (*p < 0.05). **E** Cell lysates from LRRK2-WT and -GS astrocytes were immunoprecipitated with anti-XBP1. The XBP1-bound DNA was recovered, and qRT-PCR were performed for indicated genes promoter region (upper). Expression levels of the indicated mRNAs was analyzed by real-time qPCR (lower). Data are presented as means ± SD of four independent experiments (*p < 0.05)

Next, we sought to determine the mechanism underlying attenuation of IRE1/XBP1 signaling in LRRK2-GS astrocytes. IRE1 autophosphorylation augments further oligomerization of the protein, which cooperatively stimulates its RNase module [21]. Hence, we first investigated whether IRE1 phosphorylation and oligomerization were reduced in LRRK2-GS astrocytes. In both LRRK2-WT and -GS astrocytes, IRE1 phosphorylation was induced by tunicamycin treatment, and we observed no difference between LRRK2-WT and -GS in the induction of pIRE1-containing oligomers by ER stress (Fig. 2A). Because IRE1 activation initiates splicing of XBP1 mRNA, we investigated whether splicing of XBP1 mRNA was altered in LRRK2-GS astrocytes. The translational frame-shift that converts unspliced XBP1 (XBP1u: inactivate) into spliced XBP1 (XBP1s: active) was observed in both LRRK2-WT and -GS cells under ER stress (Fig. 2B). Moreover, nuclear translocation of XBP1s was also observed in both wild-type and mutant cells (Fig. 2C), suggesting that the effect of LRRK2-GS on expression of XBP1 target genes is not because of the differences in IRE1 activation, splicing of XBP1 mRNA, or even nuclear translocation of XBP1 protein. Therefore, we hypothesized that transcriptional activity of XBP1s in the nucleus was somehow defective.

To characterize the transcriptional activity of XBP1s, we transfected LRRK2-WT and -GS astrocytes with a luciferase reporter driven by a synthetic promoter containing four tandem XBP1-binding sites. The luciferase activity of XBP1s increased > 30-fold relative to control LRRK2-WT cells, but only 17-fold in LRRK2-GS (Fig. 2D). Moreover, ChIP assays revealed that less XBP1 was bound to its target genes in LRRK2-GS than in LRRK-WT astrocytes (Fig. 2E, upper). We also confirmed that expression of target genes of XBP1 was also decreased in LRRK2-GS astrocytes, comparing with LRRK2-WT (Fig. 2E, lower). Taken together, these findings indicate that LRRK2-GS negatively regulates the transcriptional activity of XBP1s in a manner that is independent of IRE1 activation.

### XBP1s SUMOylation is elevated in LRRK2-GS astrocytes

**Fig. 3:**
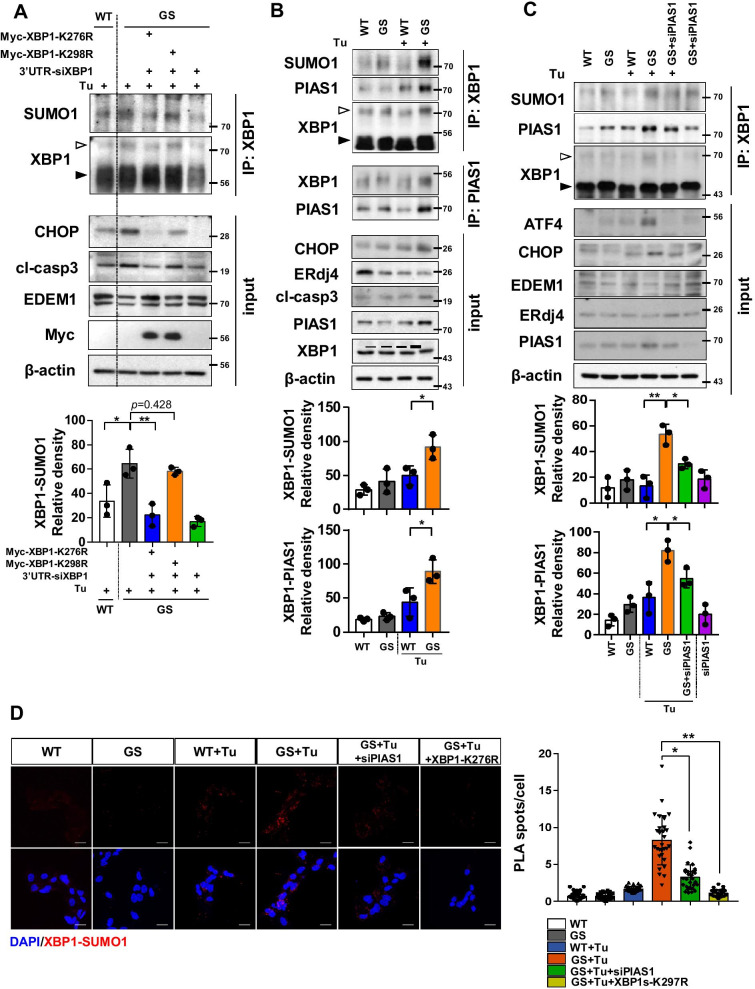
XBP1s SUMOylation increases in LRRK2-GS astrocytes. **A** Immunoprecipitation pulling down XBP1 in LRRK2-WT and -GS astrocytes co-transfected with different Myc-XBP1a mutant constructs and siRNA targeting 3ʹ-UTR region of XBP1s. SUMOylated XBP1 band intensity were quantified (lower graph). Data are means ± SD (*p < 0.05, **p < 0.01). **B** Astrocytes from LRRK2-WT and -GS were treated with tunicamycin and then subjected to immunoprecipitation with an antibody against XBP1 or PIAS1. SUMOylated XBP1 and XBP1-PIAS1 interacting band intensities were quantified (lower graph). Data are means ± SD (*p < 0.05). **C**, **D** LRRK2-WT and -GS astrocytes were transfected with PIAS1-specific siRNA (si-PIAS1) and then stimulated with tunicamycin. Protein interactions were assessed by immunoprecipitation with an antibody against XBP1 (**C**), and XBP1–SUMO1 interactions were detected using the PLA method (**D**). SUMOylated XBP1 and XBP1-PIAS1 interacting band intensity were quantified (lower graph). Data are means ± SD (*p < 0.05, **p < 0.01). PLA signals were quantified and displayed graphically as the average number of spots. Ten fields of view per group of three independent experiments (n = 30). Scale bar, 20 μm. Data are means ± SD (**p < 0.01). Open arrowhead denotes SUMOylated XBP1 and black arrowhead indicates non-modified XBP1. All band intensities were quantified using an Image J program

The activity of XBP1s is modulated by post-translational modification mediated by interactions with various partners. Phosphorylation by MAPK p38 improves nuclear translocation of XBP1, acetylation by p300 stabilizes the protein, and SUMOylation can attenuate its transcriptional activity [7, 23, 34, 56]. Given our observation that LRRK2-GS inhibited the transcriptional activity of XBP1s irrespective of its splicing and nuclear translocation, we hypothesized that the SUMOylation of XBP1s was elevated in LRRK2-GS astrocytes. To test that hypothesis, we performed immunoprecipitation assays on LRRK2-WT and LRRK2-GS astrocytes using anti-XBP1 antibody. The level of SUMOylation of XBP1 was higher in LRRK2-GS astrocytes than in LRRK2-WT cells. These SUMOylation event were abolished when SUMOylation site mutant of XBP1s (XBP1s-K276R) were introduced to the cells, whereas non-specific mutant XBP1s-K298R had no effect on XBP1s SUMOylation (Fig. 3A), consistent with previous reports [7]. In line with these results, expression level of CHOP and EDEM1 was changed depending on the status of XBP1 SUMOylation (Fig. 3A). Although PIAS2 E3 ligase was previously reported to interact with XBP1s and mediate SUMOylation of XBP1s [7], we observed that XBP1s in astrocytes interacted with PIAS1 rather than PIAS2, 3, and 4 (Additional file 1: Fig. S2A). Using siRNA targeting each PIAS, we confirmed that PIAS1 was fully responsible for SUMOylation of XBP1s in astrocytes (Additional file 1: Fig. S2B). Although other types of PIAS weakly interact with XBP1s, PIAS1 is the major SUMO E3 ligase for XBP1s in astrocytes. Hence, we investigated whether the XBP1s–PIAS1 interaction was altered in LRRK2-GS astrocytes. The immunoprecipitation data revealed that the XBP1s–PIAS1 interaction was stronger in LRRK2-GS than in LRRK2-WT astrocytes (Fig. 3B). In LRRK2-GS astrocytes transfected with PIAS1-specific siRNA, the XBP1s–SUMO interaction was diminished, and expression of XBP1 target proteins was elevated relative to the control (Fig. 3C). This result was further confirmed by proximity ligation assay (PLA), which detects proteins located within a radius of < 40 nm (Fig. 3D). Taken together, these results suggest that PIAS1 promotes XBP1s SUMOylation, thereby decreasing XBP1 transcriptional activity in LRRK2-GS astrocytes.

### SHP contributes to SUMOylation of XBP1s by increasing PIAS1 protein stability

**Fig. 4:**
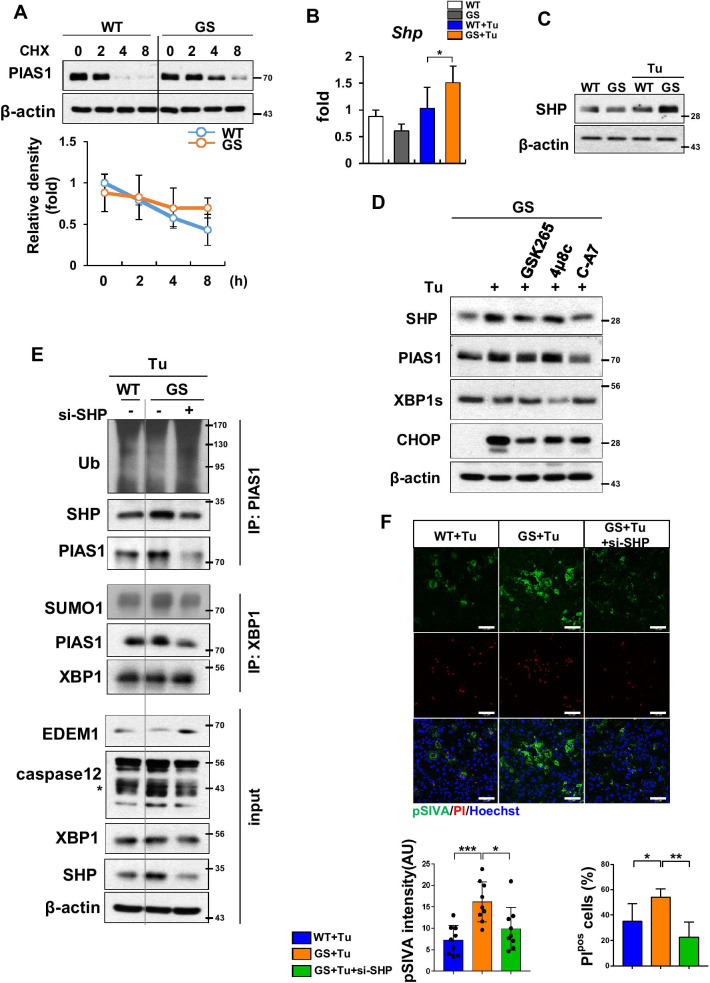
SHP regulates XBP1s SUMOylation through increasing PIAS1 protein stability. **A** Immunoblotting for PIAS1 in LRRK2-WT and -GS astrocytes treated with cycloheximide (CHX; 100 mg/ml) for up to 8 h. Data are means ± SD of three independent experiments (*p < 0.05). **B**, **C** Expression levels of SHP mRNA (**B**) and proteins (**C**) were assessed by real-time qPCR and WB, respectively. **D** LRRK2-GS astrocytes were treated with tunicamycin in the presence or absence of PERK kinase inhibitor (GSK2656157), IRE1 inhibitor (4μ8c), or ATF6 inhibitor (Ceapin-A7). Expression levels of indicated proteins were analyzed by WB. **E**, **F** LRRK2-WT and si-SHP–transfected LRRK2-GS astrocytes were treated with tunicamycin and then subjected to immunoprecipitation assay (**E**) or live/dead assay (**F**). Three fields of view per group of three independent experiments (n = 9). Scale bar, 100 μm. Data are means ± SD of three independent experiments (*p < 0.05, **p < 0.01)

Previously, we reported that SHP regulates the protein stability of PIAS1 in astrocytes by inhibiting binding of a PIAS1-specific E3 ubiquitin ligase, SIAH2 [25]. In addition, SHP actively inhibits ubiquitination of not only PIAS1 but TRAF6, a NF-κB upstream signaling molecule [60]. Thus, based on previous reports and our current results, we hypothesized that SHP plays a key role in XBP1s SUMOylation by stabilizing PIAS1 in LRRK2-GS astrocytes. To assess the stability of the PIAS1, we used a cycloheximide chasing assay. As expected, PIAS1 protein stability increased in LRRK2-GS astrocytes, although the transcript level was not altered (Fig. 4A; Additional file 1: Fig. S2C). We confirmed that there is no difference between LRRK2-WT and -GS in SHP protein stability (Additional file 1: Fig. S2D). Indeed, following tunicamycin treatment, SHP transcript and protein levels were higher in LRRK2-GS than in LRRK2-WT astrocytes (Fig. 4B, C), suggesting that up-regulation of SHP in LRRK2-GS astrocytes may be responsible for PIAS1 stabilization.

Next, we sought to determine which form of UPR signaling was involved in SHP expression under ER stress. To this end, we measured SHP protein levels in LRRK2-GS astrocytes treated with an inhibitor of PERK (GSK2656157), IRE1 (4μ8c), or ATF6 (Ceapin-A7). The results revealed that ER stress-induced SHP expression in LRRK2-GS astrocytes was reduced by inhibition of ATF6 (Fig. 4D). As with ATF6 inhibition, siRNA depletion of ATF6, but not of PERK or IRE1, decreased SHP levels (Additional file 1: Fig. S2E). This result was consistent with the findings from a previous study that ATF6 increases SHP mRNA expression in INS-1 insulinoma cell line [46]. Finally, to further assess the function of SHP in PIAS1 stability, we assessed whether ubiquitination of PIAS1 was affected by SHP knockdown. The immunoprecipitation data indicated that SHP deficiency increased PIAS1 ubiquitination in LRRK2-GS astrocytes relative to the control group (Additional file 1: Fig. S2F), suggesting that up-regulation of SHP in LRRK2-GS affects PIAS1 protein stability.

Next, we investigated whether SHP participated in SUMOylation of XBP1s. The immunoprecipitation data revealed that the absence of SHP increased ubiquitination of PIAS1 and decreased SUMOylation of XBP1s in LRRK2-GS cells (Fig. 4E). This effect of SHP on XBP1 SUMOylation was confirmed by the PLA method (Additional file 1: Fig. S2G). To define the biological consequences of SHP, we performed kinetic apoptosis assay. Apoptotic cells were frequent in LRRK2-GS astrocytes transfected with a control siRNA, but were scarce among si-SHP-transfected LRRK2-GS astrocytes (Fig. 4F). Taken together, these results suggested that elevated levels of SHP in LRRK2-GS affect PIAS1 protein stability, leading to XBP1 SUMOylation.

### LRRK2-GS astrocytes cause death of cortical neurons

**Fig. 5:**
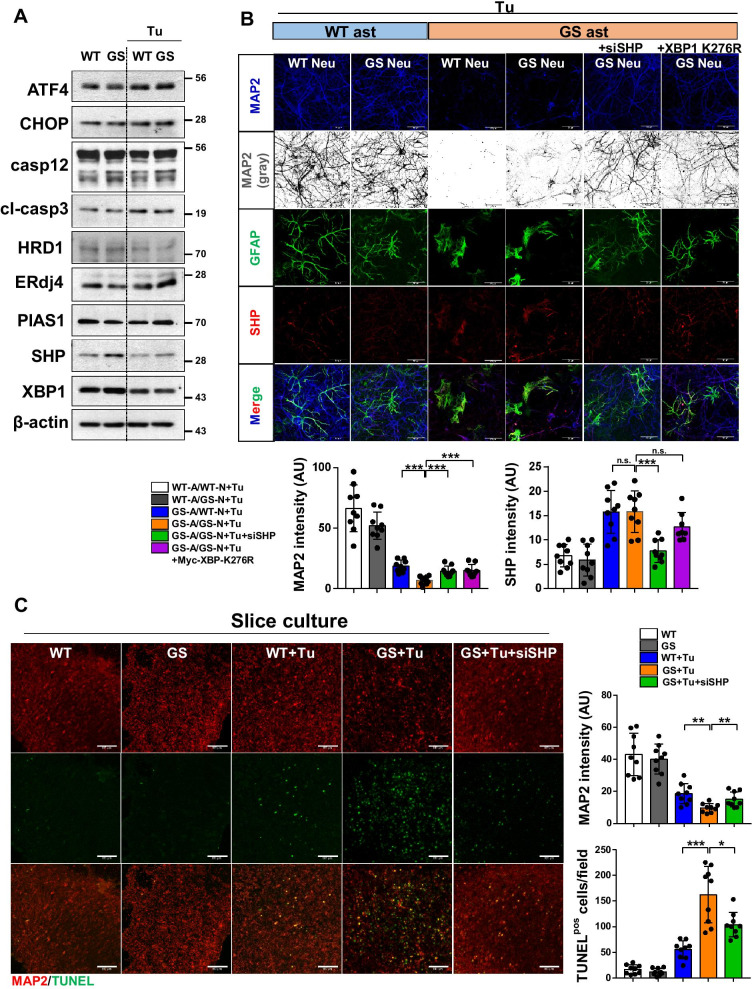
SHP is responsible for neuronal death caused by LRRK2-GS astrocytes. **A** Primary neurons isolated from LRRK2-WT and -GS mice were treated with tunicamycin, and then the indicated protein levels were analyzed by WB. **B** LRRK2-WT and -GS neurons were layered on top of LRRK2-WT or si-SHP or myc-XBP1-K276R transfected LRRK2-GS astrocytes for 7 d in vitro. Immunofluorescence staining of co-cultures was performed with indicated antibodies; representative results are shown. Summary data showing the fluorescence intensities of MAP2 and SHP per field (n = 12). Scale bar, 50 μm. Data are presented as means ± SD of three independent experiments (*p < 0.05). **C** Organotypic slices from LRRK2-WT and-GS mice were transfected with si-SHP (200 nM) for 48 h and then treated with tunicamycin (0.2 μg/ml) for 48 h. Representative images (left) and summary data (right) showing MAP2 and TUNEL staining. Five fields of view per group of three independent experiments (n = 15). Scale bar, 100 μm. Data are means ± SD (*p < 0.05, **p < 0.01)

Among the cells of the brain, astrocytes perform a variety of maintenance and regulatory functions such as regulation of glutamatergic signaling, maintenance of metabolites and extracellular ions, synaptic maintenance, and structural support [40]. Recent studies of PD-associated mutations revealed that the affected genes play functional roles in astrocyte biology, and that in several cases, the mutant phenotypes are detrimental to the surrounding neurons. We previously suggested that ER stress-induced death of LRRK2-GS astrocytes aggravates neuronal damage, and that the functions of LRRK2-GS in neurons and astrocytes differ with respect to ER stress [24]. In this study, as in our previous report, we observed no difference between LRRK2-WT and -GS neurons in expression of PERK-dependent apoptotic proteins or XBP1-induced proteins. Moreover, the expression levels of SHP were comparable in LRRK2-WT and -GS (Fig. 5A; Additional file 1: Fig. S3A). Therefore, we hypothesized that cell death due to XBP1 dysfunction in LRRK2-GS astrocytes affects neuronal survival. To determine whether LRRK2-GS astrocytes are involved in the dysfunction and loss of cortical neurons, we used a co-culture system in which LRRK2-WT or LRRK2-GS cortical neurons were directly plated onto a feeder layer of LRRK2-WT or LRRK2-GS astrocytes (Additional file 1: Fig. S3B). In LRRK2-WT as well as LRRK2-GS neurons plated on LRRK2-GS astrocytes, tunicamycin treatment decreased the abundance of MAP2 + neurons whereas there is no changes in GFAP + astrocytes (Fig. 5B; Additional file 1: Fig. S3C). Moreover, the percentage of MAP2 + neurons was reduced, whereas TUNEL-positive cells were more abundant, in brain slices from LRRK2-GS mice (Fig. 5C). When expression of SHP was depleted in LRRK2-GS cells by siRNA transfection, the frequency of cell death among neurons and astrocytes was reduced (Fig. 5B, C). In control experiments in primary neuron only, we confirmed that there is no difference between LRRK2-WT and -GS neuron in abundance of MAP2 + neuron (Additional file 1: Fig. S3D). These results confirm that the functions of LRRK2-GS in neurons and astrocytes differ with respect to ER stress. Specifically, neuronal damage and death in LRRK2-GS mice reflect the effects of abnormal astrocytes, rather than a direct effect of LRRK2-GS on neurons.

### Repression of SHP with doxycycline decreases death of LRRK2-GS astrocytes

**Fig. 6:**
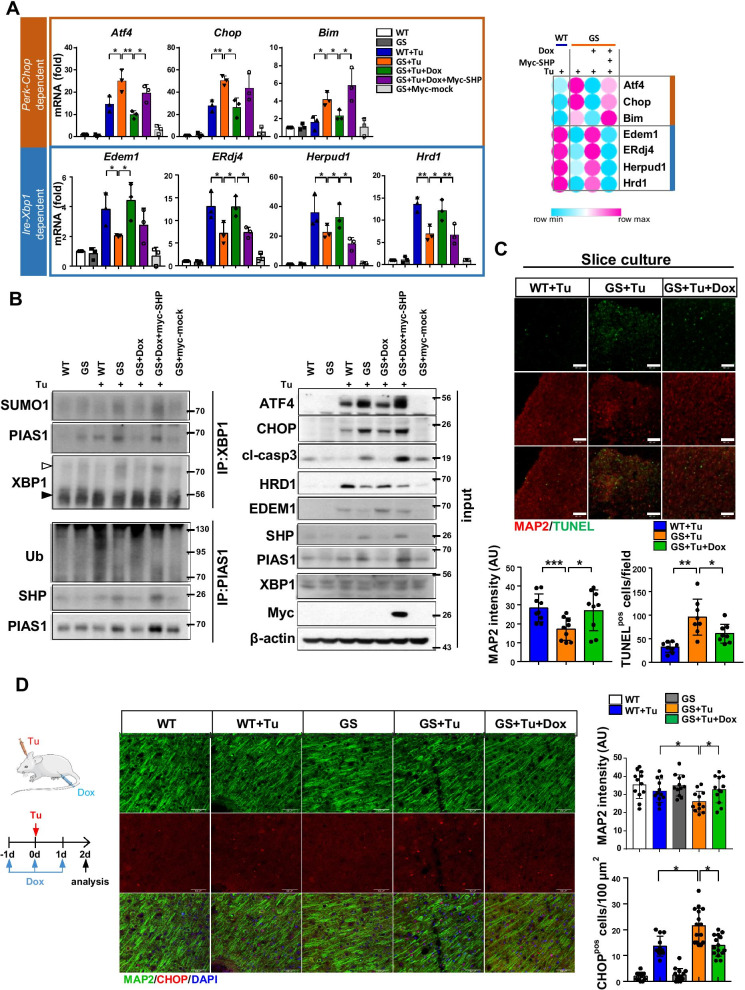
Doxycycline improves LRRK2-GS astrocyte viability by suppressing SHP expression. **A** LRRK2-WT or myc-SHP transfected LRRK2-GS astrocytes were treated with tunicamycin in the absence or presence of doxycycline (Dox) for 24 h. Expression levels of the indicated mRNAs were analyzed by real-time qPCR. **B** Immunoprecipitation pulling down XBP1 in tunicamycin-treated LRRK2-WT and -GS astrocytes in the absence or presence of doxycycline. **C** Organotypic slices from LRRK2-WT and -GS mice were treated with tunicamycin for 48 h in the absence or presence of doxycycline (50 μM). Representative images (upper) and summary data (lower) show MAP2 and TUNEL staining. Four fields of view per group of three independent experiments (n = 12). Scale bar, 100 μm. Data are means ± SD (*p < 0.05, **p < 0.01). **D** Overview of experimental timeline. Doxycycline (5 μg/g) treatment was initiated 1 d pre-injection (DMSO or tunicamycin) and then continued for the duration of the experiment. The fluorescence intensities of MAP2 per field and CHOP^pos^ cell numbers were quantified and displayed graphically. Scale bar: 50 μm. Data are means ± SD (*p < 0.05). n = 4–5/group

The results described above inspired us to search for inhibitory molecules capable of suppressing the effect of SHP on PIAS1-mediated XBP1-SUMOylation. Although no antagonist ligands have yet been identified for SHP, a previous study suggested that certain steatotic agents can inhibit SHP expression by affecting C/EBPα signaling [3]. Among those agents, we found that doxycycline decreased expression of SHP in LRRK2-GS astrocytes at a low concentration, whereas valproate (VPA) and cyclosporine A (CsA) had no effect on SHP expression (Additional file 1: Fig. S4A–C). Moreover, expression pattern of PERK-dependent and IRE1-XBP1-dependent markers in GS astrocytes were reversed by doxycycline treatment (Fig. 6A and Additional file 1: Fig. S4A–C). Inhibition of SHP by doxycycline decreased SUMOylation of XBP1 in LRRK2-GS (Fig. 6B). Furthermore, survival of LRRK2-GS astrocytes was improved by doxycycline treatment (Additional file 1: Fig. S4D). Using our neuron–astrocyte co-culture system, together with brain slice cultures of LRRK2-GS mice, we observed that increased cortical and dopaminergic neuronal death in LRRK2-GS astrocytes was attenuated by treatment with doxycycline (Fig. 6C and Additional file 1: Fig. S4E, F). These effects of doxycycline were largely reversed by SHP re-expression, suggesting the effect of doxycycline was not off-target in this regard (Fig. 6A, B). To further investigate whether doxycycline decreases severe ER stress in LRRK2-GS mice in vivo, we injected mice intraperitoneally with doxycycline prior to cortical administration of tunicamycin. In tunicamycin-injected cortex, the percentage of MAP2 + neurons was reduced, and CHOP expression was elevated relative to the vehicle-treated group. This increase in CHOP expression was attenuated in mice challenged with doxycycline (Fig. 6D), demonstrating that doxycycline effectively decreased ER stress in LRRK2-GS brain. Overall, these results suggest that doxycycline could be used to alleviate ER stress in LRRK2-GS astrocytes, as well as neuronal cell death, by disrupting SHP expression.

## Discussion

**Fig. 7:**
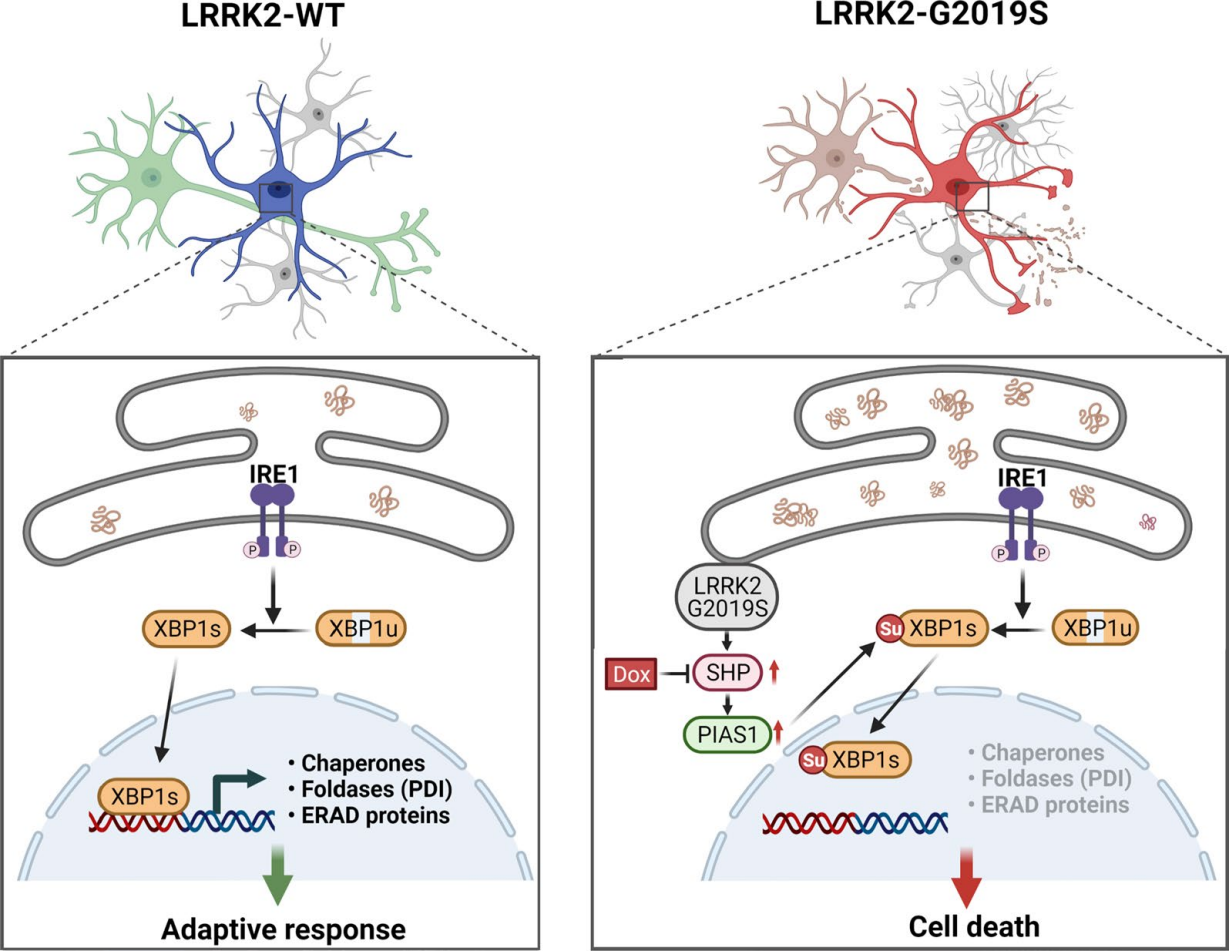
A model showing the regulatory mechanism involving SHP/PIAS1/XBP1 in LRRK2-G2019S mutant astrocytes. In the normal brain, the ER stress activates three UPR sensors, IRE1, PERK and ATF6. One of them, IRE1 activates the transcription factor XBP1 to induce genes that facilitate protein folding and removal of unfolded proteins by ER-associated degradation, including some that encode ER chaperones. However, LRRK2-G2019S negatively regulated XBP1 transcriptional activity by promoting PIAS1-mediated SUMOylation of XBP1, but did not affect phosphorylation and oligomerization of IRE1. LRRK2-G2019S increased expression of SHP, thereby stabilizing the PIAS1 protein. By increasing PIAS1 stability, SHP promotes XBP1 SUMOylation, resulting in failure of adaptive efforts and neuronal death. In this study, we identified doxycycline can disrupt SHP-mediated XBP1 SUMOylation and may therefore have therapeutic activity in PD caused by the LRRK2-GS mutation

Although the genetic mutations linked with familial PD have revealed a closer relationship between pathology and unmitigated ER stress, the underlying mechanisms remain unclear. Here, we showed that XBP1 activity was suppressed in LRRK2-GS astrocytes, resulting in failure of adaptive efforts and neuronal death. Moreover, we identified SHP as the key molecule involved in controlling XBP1 activity in LRRK2-GS astrocytes. By increasing PIAS1 stability, SHP promotes XBP1 SUMOylation, thereby driving astrocyte and neuronal death (Fig. 7).

The IRE1/XBP1 axis is associated with adaptive programs that alleviate ER stress, and thus plays a neuroprotective role. In support of this idea, expression of XBP1s using adeno-associated virus (AAV) provides outstanding neuroprotection in multiple mouse models of protein misfolding disorders (PMDs) [53]. However, recent studies suggested that XBP1 plays conflicting roles in cell survival, depending on the type of neurogenerative disease. Despite the expectation that targeting XBP1 would accelerate disease progression, conditional genetic deletion of XBP1 in the CNS protects against experimental amyotrophic lateral sclerosis (ALS) [15]. In a mouse model of Huntington’s disease (HD), one study showed that sustained activation of IRE1 can trigger neuronal loss [17], whereas expression of XBP1s decreased aggregation of mutant huntingtin in the striatum [62]. In PD mouse models, ablation of XBP1 in the substantia nigra results in chronic ER stress, triggering degeneration of dopaminergic neurons [43, 52]. These studies imply that XBP1 is an executor of cell fate determination as each disease’s circumstance. In this study, we confirmed that XBP1 signaling is indispensable for the viability of astrocytes, and we showed that transcriptional deactivation of XBP1 by LRRK2-GS mutation causes astrocyte death. Previously, we suggested that LRRK2-GS impairs ER Ca^2+^ homeostasis, which determines cell survival, and it could thus contribute to the development of PD [24]. In addition, we also revealed another mechanistic role of LRRK2-GS in the ER stress response: regulation of XBP1 transcriptional activity. A recent study showed that XBP1 activation in astrocytes play a role in neuroprotection [12], suggesting that proper regulation of XBP1 activity in astrocytes is critical for neuronal survival. Given that perturbation of astrocyte function can be detrimental to neurons, decreased XBP1 signaling in LRRK2-mutated astrocytes may contribute neuronal cell damage and PD progress. In this regard, a therapeutic strategy targeting XBP1 activity in astrocytes has the potential to ameliorate PD pathogenesis.

Previous studies show that a LRRK2-GS mouse model exhibited a modest loss of dopaminergic neurons accompanied by accumulation of autophagosomes, mitochondrial alterations, and ER stress, revealing the direct toxic effect of LRRK2-GS on neurons [20, 39, 59]. In this study, however, there was no difference in the expression of ER stress proteins and SHP or cell death between LRRK2-WT and -GS neurons. These differential effects could be due, in part, to the sensitivity to tunicamycin. Low-dose tunicamycin can activate the ER stress response in LRRK2-GS astrocytes, but may not be sufficient to activate neuronal ER stress, suggesting that astrocytes, but not neurons, may be particularly sensitive to misfolded or unfolded protein accumulation in LRRK2-GS mice. Overall, our results indicate that neuronal damage and death in LRRK2-GS mice reflect the effect of dysfunctional astrocytes, rather than a direct effect of LRRK2-GS on neurons. Recent studies and our previous study also emphasized the role of astrocytic UPR activation in neurodegenerative disease, rather than direct neuronal, cell-autonomous effects of ER stress [12, 24, 48].

Members of the nuclear receptor (NR) superfamily of ligand-dependent transcription factors play diverse roles in development, homeostasis, reproduction, and immunity [5, 6]. Recent work showed that NRs contribute to ER stress resolution and ER stress-mediated apoptosis in various metabolic tissues. For example, liver receptor homolog-1 (LRH-1) activation improves type 2 diabetes mellitus by helping to resolve ER stress [27]. Similarly, the farnesoid X receptor (FXR) signaling pathway induced by ER stress ameliorates ER stress-mediated nonalcoholic fatty liver disease (NAFLD) [57]. Agonists for peroxisome proliferator-activated receptor (PPAR)α/γ exert antidiabetic effects associated with improving ER stress in metabolic tissues [50, 58]. Treatment with agonists of liver X receptors (LXRs) decrease saturated fatty acid-induced ER stress in primary hepatocytes [41]. However, the role of SHP in ER stress and cell death remains controversial. One study reported that SHP mediates ATF6-induced β-cell dysfunction [46], whereas another study suggested that SHP is associated with FXR agonist-induced activation of the hepatic IRE1/XBP1 pathway, thereby promoting cell survival [26]. Intriguingly, Sun and colleagues suggested that SHP physically interacts with XBP1s, thereby inhibiting its polyubiquitination and degradation [51]. In LRRK2-GS astrocytes, however, neither the SHP–XBP1 interaction nor SHP-mediated XBP1 ubiquitination differed between LRRK2-WT and -GS (Additional file 1: Fig. S5), suggesting that the effects of SHP on ER stress may vary among cell types and contexts. In brain astrocytes, we observed that expression of SHP increased in LRRK2-GS astrocytes, and that SHP dynamically regulated the transcriptional activity of XBP1 via PIAS1-mediated SUMOylation, leading to unresolved ER stress and cell death. Moreover, the effect of SHP on ER stress was specific for astrocytes, not neurons. Given that perturbation in astrocyte functions can be detrimental to neurons, unraveling the specific role of SHP in LRRK2-mutated astrocytes is of potential clinical importance. In turn, our findings suggest that a therapeutic strategy targeting SHP in astrocytes has the potential to ameliorate PD pathogenesis.

Many drugs used to treat PD are of limited effect due to their toxicity or low bioavailability in the brain. This situation has inspired the exploration of alternative drug strategies, such as repurposing existing compounds with novel targets. In this study, we found that doxycycline has the potential to prevent ER stress in LRRK2-GS astrocytes by targeting SHP. Doxycycline, a second-generation tetracycline, is commonly used as an antibiotic in humans, and the drug is both clinically safe and capable of penetrating the blood–brain barriers. Because long-term administration (up to 2 years) of sub-antimicrobial doses of doxycycline (20 mg twice daily) has not produced antibiotic side effects in clinical trials [19, 36], it is an attractive candidate for drug repurposing to treat CNS diseases. In fact, along with its well-characterized effects, doxycycline can protect against neuronal cell death by suppressing expression of matrix metalloproteinases (MMPs) and activation of glial cells, reducing the load of Aβ oligomers (AβOs), remodeling α-synuclein early aggregates, and scavenging ROS [2, 13, 18, 22]. Hence, doxycycline may help to establish strategies for novel treatments of neurodegenerative disorders. However, the mechanisms underlying its benefits remain unclear. Here, we propose an additional role of doxycycline in the ER stress response. We found that a low dose of doxycycline (10 μM) inhibited the expression of SHP, thereby eliciting the XBP-1-mediated adaptive ER stress response, in LRRK2-GS mutated astrocytes (Fig. 6).

Although doxycycline is a promising candidate for treatment of various neurodegenerative diseases, it has yielded variable and even contradictory (i.e., beneficial and detrimental) effects in various species and models [1, 11, 37, 47]. Even in the ER stress response, the effect of doxycycline is not clear: Matsumoto and colleagues showed that doxycycline induces ER stress and apoptosis in sphere-forming cells (i.e., cancer stem-like cells) [29], whereas we showed that the severe ER stress response was reduced by doxycycline in brain astrocytes. Accordingly, targeting ER stress using doxycycline should be considered based on the specific cellular milieu and type of neurodegenerative disorder. Here, we suggest that doxycycline may selectively apply to the PD-related LRRK2-G2019S mutation model. Additional studies are required to determine whether sub-antibiotic doses of doxycycline are safe for PD patients during prolonged treatments, as well as whether combination therapy with doxycycline and existing treatments for PD (e.g., LRRK2 kinase inhibitors) represent potential therapeutic approaches.

## Conclusion

Here, we report a novel mechanism by which the PD-associated LRRK2-G2019S mutant accelerates ER stress in brain astrocytes. We show that LRRK2-G2019S negatively regulated XBP1 transcriptional activity by promoting PIAS1-mediated XBP1 SUMOylation. Intriguingly, we found that XBP1 SUMOylation in LRRK2-G2019S astrocytes was mediated by the orphan nuclear receptor SHP. Our data reveal a novel post-translational mechanism for regulation of XBP1 and establish the importance of SHP in modulating ER homeostasis in PD pathogenesis. These findings provide important insight into the basis of the correlation between mutant LRRK2 and pathophysiological ER stress in PD, and suggest a plausible model that explains this connection.

## Supplementary Information

**Additional file 1: Figure S1.** Rel Fig. 1. XBP1 inactivation on brain astrocytes. **Figure S2.** Rel Figs. 3 and 4. Effect of SHP on XBP1 SUMOylation in LRRK2-GS astrocytes. **Figure S3.** Rel Fig. 5. The ER stress response in LRRK2 mutated neurons. **Figure S4.** Rel Fig. 6. Effect of doxycycline on SHP-mediated XBP1 activity. **Figure S5.** Effect of SHP on XBP1 ubiquitination. **Table S1. **Comparison of genes expression in LRRK2-WT and -GS astrocytes. **Table S2. **List of primer sequences. **Table S3. **List of siRNA oligonucleotides. **Table S4. **List of primary antibodies used in this work.

## Data Availability

All data relevant to the study are included in the article or as supplementary information. Upon reasonable request, additional information (e.g., protocols) will be shared by the corresponding authors.

## Abbreviations

PD: Parkinson’s disease
LRRK2-GS: LRRK2-Gly2019Ser
ER stress: Endoplasmic reticulum stress
PERK: PKR-like endoplasmic reticulum kinase
IRE1: Inositol-requiring enzyme 1
ATF6: Activating transcription factor 6
XBP1: X-box protein 1
PIAS1: Protein inhibitor of activated STAT 1
SHP: Small heterodimer partner
Dox: Doxycycline
