# Supplementary material for: Small heterodimer partner (SHP) aggravates ER stress in Parkinson’s disease-linked LRRK2 mutant astrocyte by regulating XBP1 SUMOylation
Source: J Biomed Sci. 2021 Jul 7;28:51. doi: 10.1186/s12929-021-00747-1 (PMC8261914; doi:10.1186/s12929-021-00747-1)
Supplement: Supplementary file 1 — Additional file 1: Figure S1. Rel Fig. 1. XBP1 inactivation on brain astrocytes. Figure S2. Rel Figs. 3 and 4. Effect of SHP on XBP1 SUMOylation in LRRK2-GS astrocytes. Figure S3. Rel Fig. 5. The ER stress response in LRRK2 mutated neurons. Figure S4. Rel Fig. 6. Effect of doxycycline on SHP-mediated XBP1 activity. Figure S5. Effect of SHP on XBP1 ubiquitination. Table S1. Comparison of genes expression in LRRK2-WT and -GS astrocytes. Table S2. List of primer sequences. Table S3. List of siRNA oligonucleotides. Table S4. List of primary antibodies used in this work. [file 12929_2021_747_MOESM1_ESM.pdf]

Supplemental Materials

Small heterodimer partner (SHP) aggravates ER stress in the Parkinson's disease-linked LRRK2 mutant astrocyte by regulating XBP1 SUMOylation

Jee Hoon Lee, Ji-hye Han, Eun-hye Joe and Ilo Jou*

The supplementary material comprises figures S1-S5 and supplementary tables 1-4.

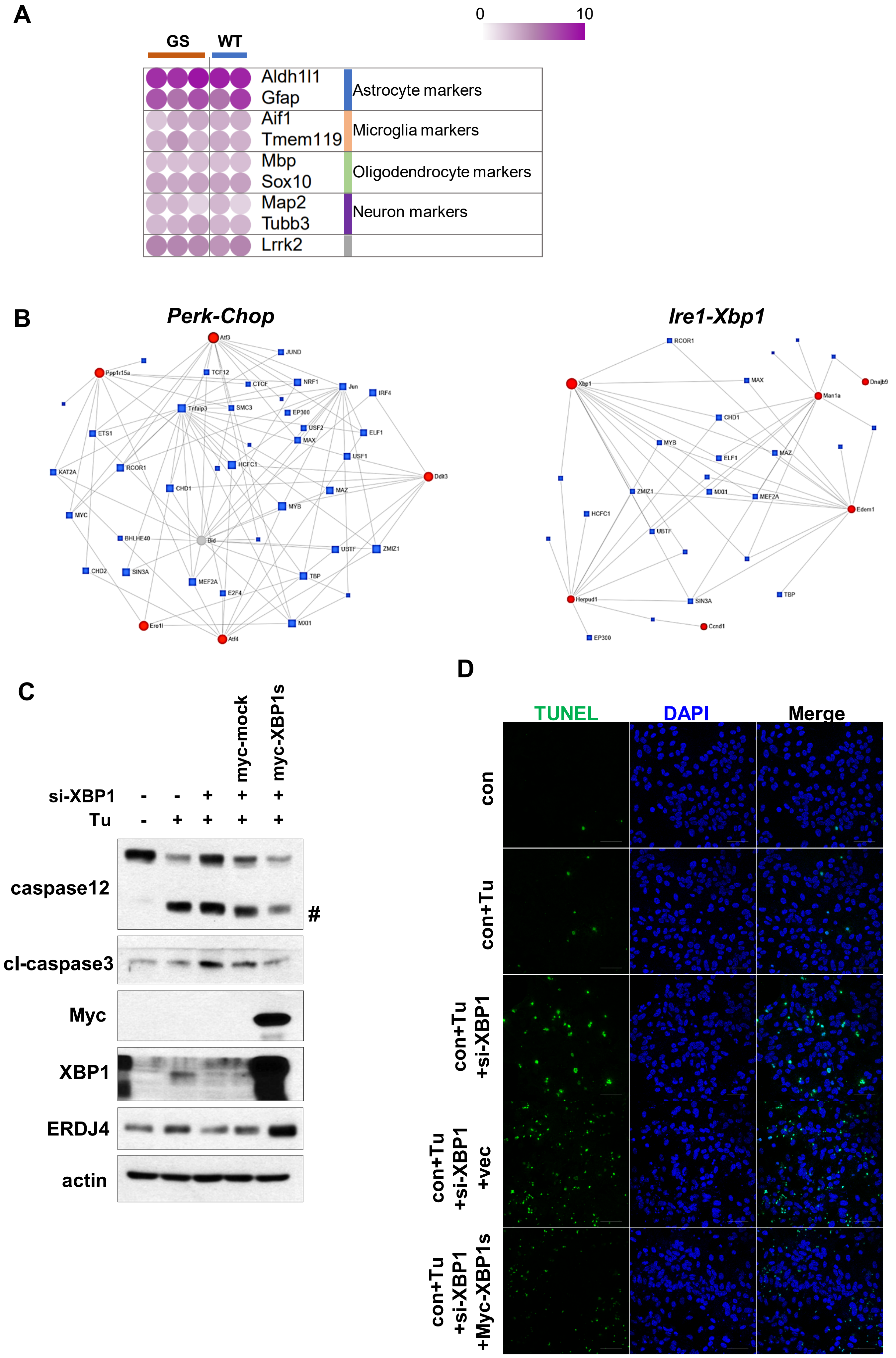

A
GS
WT
0
10
Aldh1l1
Gfap
Astrocyte markers
Aif1
Tmem119
Microglia markers
Mbp
Sox10
Oligodendrocyte markers
Map2
Tubb3
Neuron markers
Lrrk2
B
Perk-Chop
Ire1-Xbp1
C
si-XBP1
Tu
myc-mock
myc-XBP1s
caspase12
#
cl-caspase3
Myc
XBP1
ERDJ4
actin
D
TUNEL
DAPI
Merge
con
con+Tu
con+Tu +si-XBP1
con+Tu +si-XBP1 +vec
con+Tu +si-XBP1 +Myc-XBP1s

Figure S1, Rel Figure 1. XBP1 inactivation on brain astrocytes.

(**A**) Heatmap from DNA microarray analysis of LRRK2-WT and –GS astrocytes showing gene expression level of well described markers for astrocytes (Glial fibrillary acidic protein; *Gfap*, Aldehyde Dehydrogenase 1 Family Member L1; *Aldh1l1*), microglia (Allograft inflammatory factor 1; *Aif1*, Transmembrane Protein 119; *Tmem119*), oligodendrocyte (Myelin Basic Protein; *Mbp*, SRY-Box Transcription Factor 10; *Sox10*), and neuron (microtubule-associated protein 2; *Map2*, Tubulin Beta 3 Class III; *Tubb3*). The gene expression level of LRRK2 was also described. **(B)** Pathway analysis of genes involved in the ER stress response that were identified by microarray profiling. The network diagram was created with NetworkAnalyst (http://www.networkanalyst.ca). **(C and D)** Primary astrocytes were co-transfected with siRNA targeting the 3'-UTR region of XBP1 and/or Myc-tagged XBP1s. After 48 h, cells were stimulated with tunicamycin, and the expression levels of indicated proteins and cell viability were analyzed by WB (C) and TUNEL assay (D), respectively. Scale bar, 50 μm.

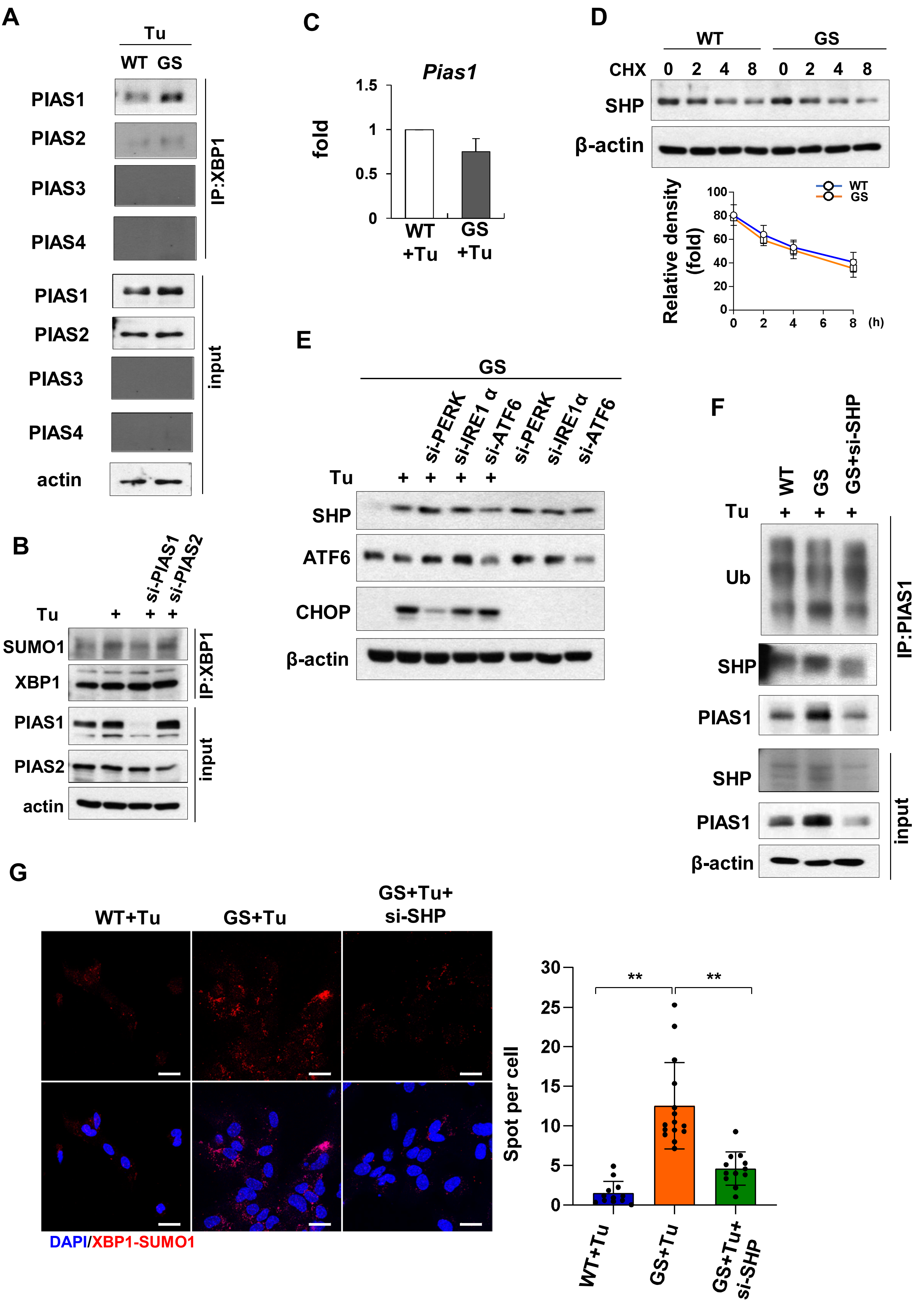

A
Tu
WT GS
PIAS1
PIAS2
PIAS3
PIAS4
IP:XBP1
PIAS1
PIAS2
PIAS3
PIAS4
actin
input
B
Tu + + +
si-PIAS1
si-PIAS2
SUMO1
XBP1
IP:XBP1
PIAS1
PIAS2
actin
input
C
Pias1
fold
1.5
1
0.5
0
WT +Tu
GS +Tu
D
WT
GS
CHX 0 2 4 8 0 2 4 8
SHP
β-actin
Relative density (fold)
100
80
60
40
20
0
0 2 4 6 8 (h)
WT
GS
E
GS
si-PERK
si-IRE1 α
si-ATF6
si-PERK
si-IRE1α
si-ATF6
Tu + + + +
SHP
ATF6
CHOP
β-actin
F
WT
GS
GS+si-SHP
Tu + + +
Ub
SHP
PIAS1
IP:PIAS1
SHP
PIAS1
β-actin
input
G
WT+Tu
GS+Tu
GS+Tu+ si-SHP
DAPI/XBP1-SUMO1
Spot per cell
30
25
20
15
10
5
0
**
**
WT+Tu
GS+Tu
GS+Tu+ si-SHP

Figure S2, Rel Figure 3 and 4. Effect of SHP on XBP1 SUMOylation in LRRK2-GS astrocytes.

(A) Astrocytes isolated from LRRK2-WT and -GS mice were treated with tunicamycin, and protein interactions were measured by immunoprecipitation with antibody against XBP1. **(B)** si-PIAS1– or si-PIAS2–transfected LRRK2-GS astrocytes were treated with tunicamycin and then subjected to immunoprecipitation assay. **(C)** PIAS1 transcript level in tunicamycin-treated LRRK2-WT and -GS astrocytes. **(D)** Immunoblotting for SHP in LRRK2-WT and -GS astrocytes treated with cycloheximide (CHX; 100 mg/ml) for up to 8 h. Data are means ± SD of three independent experiments. **(E)** LRRK2-GS astrocytes were transfected with siRNA against PERK, IRE1, or ATF6 for 48 h and then treated with tunicamycin. Expression of the indicated proteins was analyzed by WB. **(F and G)** LRRK2-WT and si-SHP transfected LRRK2-GS astrocytes were treated with tunicamycin, and cells were subjected to immunoprecipitation assay (F) and PLA assay (G). Five fields of view per group of three independent experiments (n = 15). Scale bar, 20 µm. Data are presented as means ± SD (***p* < 0.01).

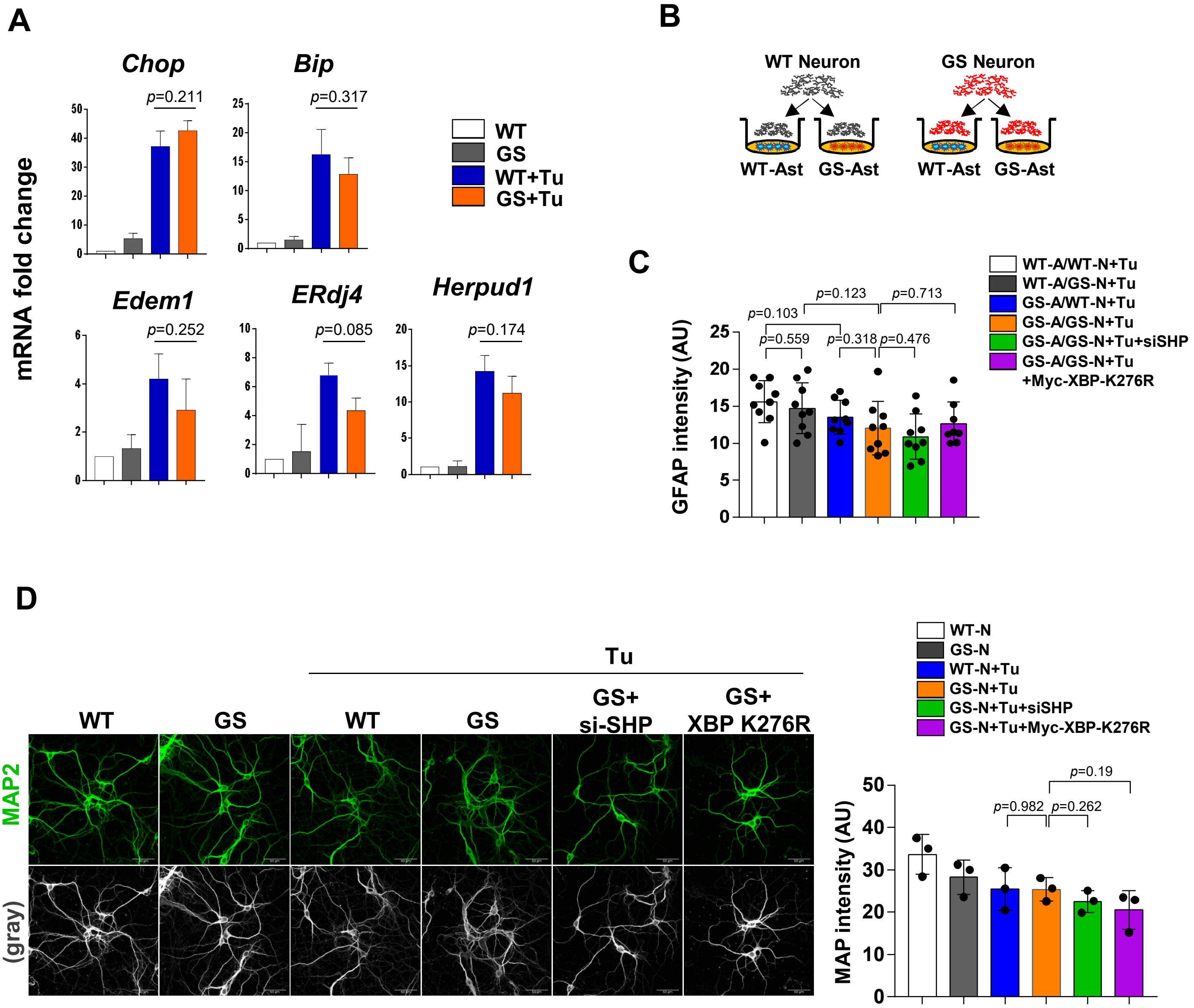

Figure S3, Rel Figure 5. The ER stress response in LRRK2 mutated neurons.

(A) Primary neurons isolated from LRRK2-WT and -GS mice were treated with tunicamycin, and then the expression levels of the indicated mRNAs were analyzed by real-time qPCR. **(B)** Schematic depiction of co-culture experiments. **(C)** LRRK2-WT and -GS neurons were layered on top of LRRK2-WT or si-SHP or myc-XBP1-K276R transfected LRRK2-GS astrocytes for 7 days *in vitro*. Summary data showing the fluorescence intensities of GFAP per field (n = 5). Data are presented as means ± SD. **(D)** Primary neuron from LRRK2-WT and -GS were transfected with si-SHP or myc-XBP1-K276R. Representative images and summary data showing MAP2 staining. Scale bar, 50 μm. Data are presented as means ± SD.

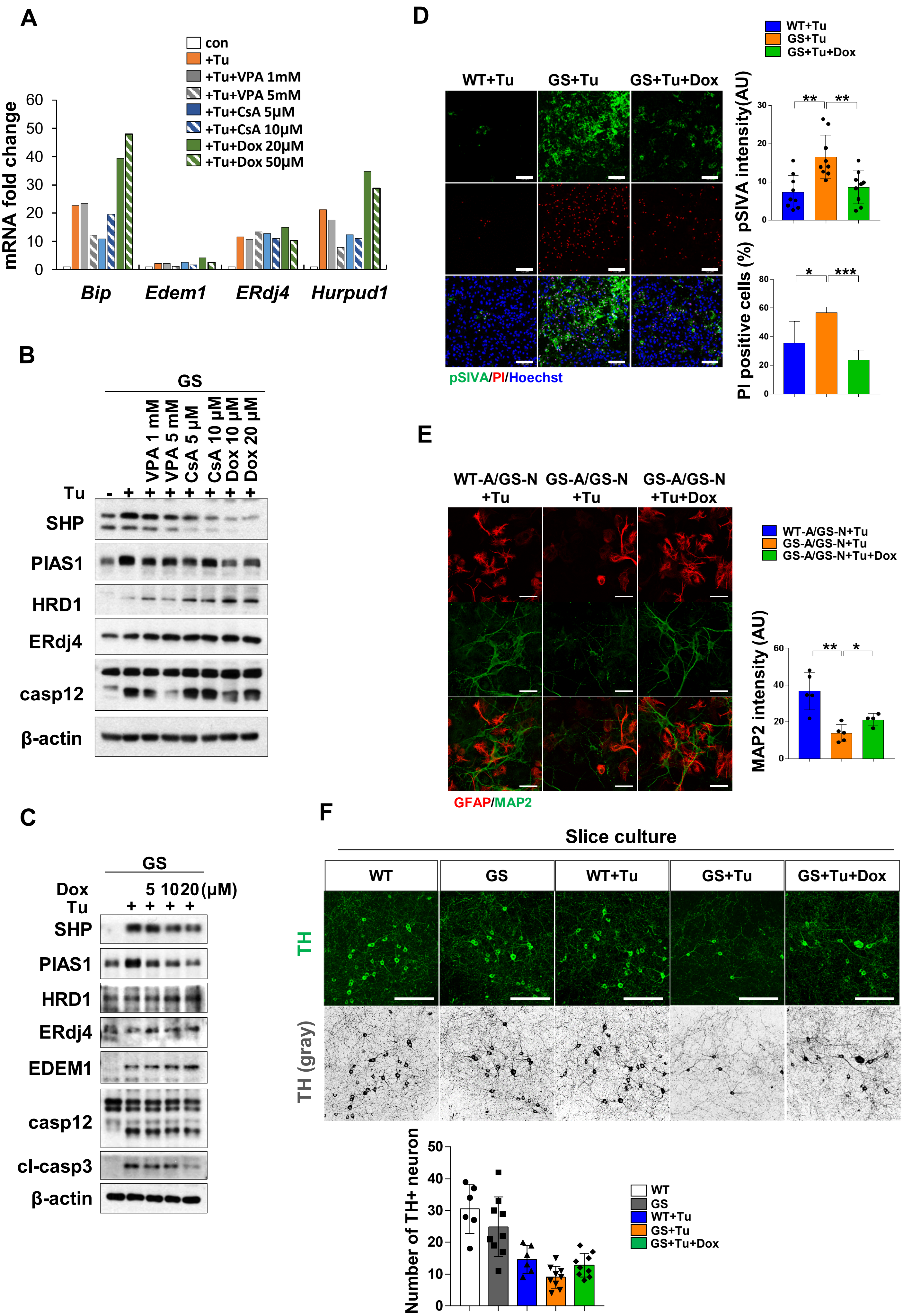

A
mRNA fold change
con
+Tu
+Tu+VPA 1mM
+Tu+VPA 5mM
+Tu+CsA 5μM
+Tu+CsA 10μM
+Tu+Dox 20μM
+Tu+Dox 50μM
Bip
Edem1
ERdj4
Hurpud1
B
GS
Tu
VPA 1 mM
VPA 5 mM
CsA 5 μM
CsA 10 μM
Dox 10 μM
Dox 20 μM
SHP
PIAS1
HRD1
ERdj4
casp12
β-actin
C
GS
Dox 5 10 20 (μM)
Tu
SHP
PIAS1
HRD1
ERdj4
EDEM1
casp12
cl-casp3
β-actin
D
WT+Tu
GS+Tu
GS+Tu+Dox
pSIVA/PI/Hoechst
pSIVA intensity(AU)
PI positive cells (%)
E
WT-A/GS-N +Tu
GS-A/GS-N +Tu
GS-A/GS-N +Tu+Dox
WT-A/GS-N+Tu
GS-A/GS-N+Tu
GS-A/GS-N+Tu+Dox
MAP2 intensity (AU)
GFAP/MAP2
F
Slice culture
WT
GS
WT+Tu
GS+Tu
GS+Tu+Dox
TH
TH (gray)
Number of TH+ neuron

Figure S4, Rel Figure 6. Effect of doxycycline on SHP-mediated XBP1 activity.

(A and B) LRRK2-GS astrocytes were treated with tunicamycin in the absence or presence of the indicated drugs, and expression of the indicated genes (A) and proteins (B) was analyzed. **(C)** LRRK2–GS astrocytes treated with tunicamycin in the presence of indicated dose of doxycycline. **(D)** Representative images and summary data showing live/dead staining of LRRK2-WT and -GS astrocytes 72 h after treatment with tunicamycin in the absence or presence of doxycycline. Five fields of view per group of three independent experiments (n = 15). Scale bar, 100 μm. Data are means ± SD (*$p < 0.05$, **$p < 0.01$). **(E)** LRRK2-GS neurons were layered on top of LRRK2-WT and LRRK2-GS astrocytes for 7 days *in vitro*, and then treated with tunicamycin in the absence or presence of doxycycline. Representative immunofluorescence staining of co-cultures with anti-GFAP and MAP2 antibodies. Summary data showing fluorescence intensities of MAP2 per cell (n = 12). Scale bar, 50 μm. (*$p < 0.05$, **$p < 0.01$). VPA, valproate; CsA, cyclosporine A; Dox, doxycycline. **(F)** Organotypic slices from LRRK2-WT and-GS mice were treated with tunicamycin for 48 h in the absence or presence of doxycycline (50 μM). Representative images and summary data show tyrosine hydroxylase (TH)-positive dopaminergic neurons. The number of TH-positive neurons was analyzed by Cytation 5 Cell Imaging Multimode Reader. Three fields of view per group of two (WT) or three (GS) independent experiments. Scale bar, 200 μm.

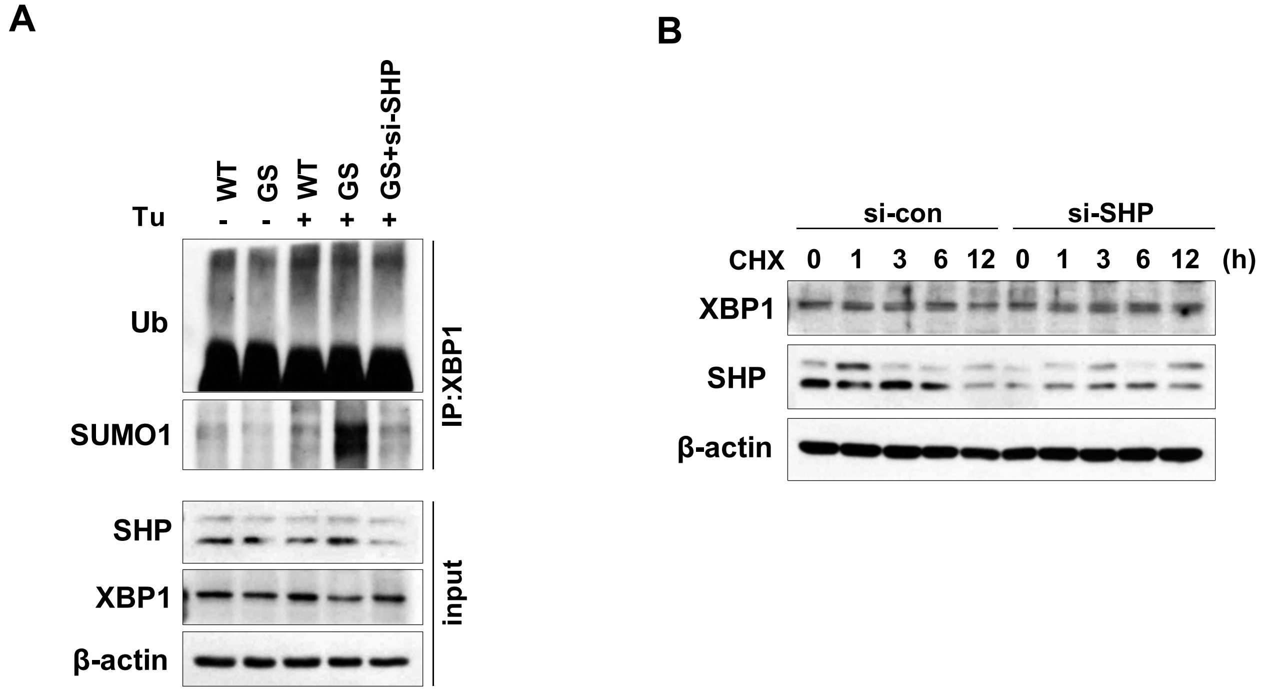

Figure S5. Effect of SHP on XBP1 ubiquitination

(A) Western blot analysis of immunoprecipitates of XBP1 in LRRK2-WT and -GS astrocytes showing XBP1 ubiquitination. Cells were treated with proteasomal inhibitor MG132 for 1 h prior to immunoprecipitation. **(B)** Immunoblotting for XBP1s in LRRK2-WT and -GS astrocytes treated with cycloheximide for up to 12 h.

Table S1. Comparison of genes expression in LRRK2-WT and -GS astrocytes

**Response to unfolded protein and ERAD**			
GeneID	Symbol	Gene Name	ration GS/WT
12443	Ccnd1	cyclin D1	-2.376412
27362	Dnajb9	DnaJ (Hsp40) homolog, subfamily B, member 9	-1.500352
192193	Edem1	ER degradation enhancer, mannosidase alpha-like 1	-1.500082
64209	Herpud1	homocysteine-inducible, endoplasmic reticulum stress-inducible, ubiquitin-like domain member 1	-1.511164
17155	Man1a	mannosidase 1, alpha	-1.624573
11910	Atf3	activating transcription factor 3	2.679428
11911	Atf4	activating transcription factor 4	1.530428
13198	Ddit3	DNA-damage inducible transcript 3	2.530259
50527	Ero1l	ERO1-like (S. cerevisiae)	1.532051
16476	Jun	jun proto-oncogene	1.515125
17872	Ppp1r15a	protein phosphatase 1, regulatory (inhibitor) subunit 15A	1.699380

**Apoptosis/Cell death**			
GeneID	Symbol	Gene Name	ration GS/WT
11606	Agt	angiotensinogen (serpin peptidase inhibitor, clade A, member 8)	-1.631889
407824	BC020402	cDNA sequence BC020402	-1.501200
64209	Herpud1	homocysteine-inducible, endoplasmic reticulum stress-inducible, ubiquitin-like domain member 1	-1.511164
11461	Actb	actin, beta	1.577261
11911	Atf4	activating transcription factor 4	1.530428
12122	Bid	BH3 interacting domain death agonist	1.949184
13198	Ddit3	DNA-damage inducible transcript 3	2.530259
50527	Ero1l	ERO1-like (S. cerevisiae)	1.532051
68680	Fitm1	fat storage-inducing transmembrane protein 1	1.740313
14281	Fos	FBJ osteosarcoma oncogene	1.848786
23882	Gadd45g	growth arrest and DNA-damage-inducible 45 gamma	1.529889
16176	Il1b	interleukin 1 beta	1.607761
16476	Jun	jun proto-oncogene	1.515125
27279	Tnfrsf12a	tumor necrosis factor receptor superfamily, member 12a	2.023498
22146	Tuba1c	tubulin, alpha 1C	1.505448

**Transmembrane transporter activity**			
GeneID	Symbol	Gene Name	ration GS/WT
11606	Agt	angiotensinogen (serpin peptidase inhibitor, clade A, member 8)	-1.631889
11732	Ank	progressive ankylosis	-1.725200
54140	Avpr1a	arginine vasopressin receptor 1A	-1.711004
13867	Erbb3	v-erb-b2 erythroblastic leukemia viral oncogene homolog 3 (avian)	-1.541481
384783	Irs2	insulin receptor substrate 2	-1.854050
26399	Map2k6	mitogen-activated protein kinase kinase 6	-1.744905
20511	Slc1a2	solute carrier family 1 (glial high affinity glutamate transporter), member 2	-1.525299
66972	Slc25a23	solute carrier family 25 (mitochondrial carrier; phosphate carrier), member 23	-1.515468
320541	Slc35e2	solute carrier family 35, member E2	-1.744562
28250	Slco1a4	solute carrier organic anion transporter family, member 1a4	-2.671466
14664	Slc6a9	solute carrier family 6 (neurotransmitter transporter, glycine), member 9	1.502470
216227	Slc17a8	solute carrier family 17 (sodium-dependent inorganic phosphate cotransporter), member 8	1.783501

Continue..

Amino acid biosynthesis/Translation/Amino acid metabolism			
GeneID	Symbol	Gene Name	ration GS/WT
353172	Gars	glycyl-tRNA synthetase	1.512909
107045	Lars	leucyl-tRNA synthetase	2.151799
70223	Nars	asparaginyl-tRNA synthetase	1.757679
14629	Gclc	glutamate-cysteine ligase, catalytic subunit	1.798725
107272	Psat1	phosphoserine aminotransferase 1	1.572591
50493	Txnrd1	thioredoxin reductase 1	1.588293
**Lipid biosynthesis/metabolism**			
GeneID	Symbol	Gene Name	ration GS/WT
26874	Abcd2	ATP-binding cassette, sub-family D (ALD), member 2	-2.225226
11606	Agt	angiotensinogen (serpin peptidase inhibitor, clade A, member 8)	-1.631889
54140	Avpr1a	arginine vasopressin receptor 1A	-1.711004
54326	Elovl2	elongation of very long chain fatty acids (FEN1/Elo2, SUR4/Elo3, yeast)-like 2	-1.550826
76267	Fads1	fatty acid desaturase 1	-1.644752
384783	Irs2	insulin receptor substrate 2	-1.854050
217166	Nr1d1	nuclear receptor subfamily 1, group D, member 1	-2.057525
18604	Pdk2	pyruvate dehydrogenase kinase, isoenzyme 2	-1.502571
18984	Por	P450 (cytochrome) oxidoreductase	-1.547853
19017	Ppargc1a	peroxisome proliferative activated receptor, gamma, coactivator 1 alpha	-1.980796
26897	Acot1	acyl-CoA thioesterase 1	1.646822
171210	Acot2	acyl-CoA thioesterase 2	1.523914
74147	Ehhadh	enoyl-Coenzyme A, hydratase/3-hydroxyacyl Coenzyme A dehydrogenase	1.839306
20280	Scp2	sterol carrier protein 2, liver	1.752942
**Toll-like receptor signaling pathway**			
GeneID	Symbol	Gene Name	ration GS/WT
14281	Fos	FBJ osteosarcoma oncogene	1.848786
16176	Il1b	interleukin 1 beta	1.607761
54123	Irf7	interferon regulatory factor 7	1.842334
16476	Jun	jun proto-oncogene	1.515125
**TNF signaling pathway**			
GeneID	Symbol	Gene Name	ration GS/WT
11911	Atf4	activating transcription factor 4	1.530428
20296	Ccl2	chemokine (C-C motif) ligand 2	1.513550
14825	Cxcl1	chemokine (C-X-C motif) ligand 1	1.543822
14281	Fos	FBJ osteosarcoma oncogene	1.848786
16176	Il1b	interleukin 1 beta	1.607761
16476	Jun	jun proto-oncogene	1.515125
21929	Tnfaip3	tumor necrosis factor, alpha-induced protein 3	1.505247

Continue..

IL-1 beta production			
GeneID	Symbol	Gene Name	ration GS/WT
74155	Errfi1	ERBB receptor feedback inhibitor 1	1.536976
14870	Gstp1	glutathione S-transferase, pi 1	3.685141
16176	Il1b	interleukin 1 beta	1.607761
21929	Tnfaip3	tumor necrosis factor, alpha-induced protein 3	1.505247
**Glutathione metabolism**			
GeneID	Symbol	Gene Name	ration GS/WT
14629	Gclc	glutamate-cysteine ligase, catalytic subunit	1.798725
14862	Gstm1	glutathione S-transferase, mu 1	1.501338
14865	Gstm4	glutathione S-transferase, mu 4	1.520065
14870	Gstp1	glutathione S-transferase, pi 1	3.685141

Table S2. List of primer sequences

Primers	Forward	Reverse
**Atf4**	TCGATGCTCTGTTTCGAATG	GGCAACCTGGTCGACTTTTA
**Chop**	GCATGAAGGAGAAGGAGCAG	CTTCCGGAGAGACAGACAGG
**Gadd34**	GCTGGGTCCTTACCTTACCC	AGGGAGTGGTCACATCTTGG
**Bim**	TCCGTCTGGTATGGAGAAGG	ACATCGACACAGTGCAGAGC
**Edem1**	TGGGCTGGATTCCTTCTATG	GGTGGGTCTCCTTCTCCTTC
**ERdj4**	GCAATGGGAGTCCTTTTGAA	CCTGGAAGTGATGCCTTTGT
**Herpud1**	ACAAAGGGTGCTGAATCCAC	CCTTGGAAAGTCTGCTGGAC
**Hrd1**	CAACTGGGGCCTTCAAAGTA	GTCTGTCTTCAGCCCCTCTG
**Pias1**	ATCAGGTAGCGTCCCACAAC	CGAGGCTTGATGAGGAAGAC
**Shp**	AGCTGGGTCCCAAGGAGTAT	CTTGAGGGTAGAGGCCATGA
**Xbp1**	ACACGCTTGGGAATGGACAC	CCATGGGAAGATGTTCTGGG
**ChIP Bip**	CATTGGTGGCCGTTAAGAAT	TGAAGTCGCTACTCGTTGGA
**ChIP Hrd1**	TGGAGGAGTTTAGGGTGGTG	GGAGTGGACCGTACCAAGTT
**ChIP Edem1**	TTCAGGGACACCATTTACCC	GGTTCCGAGTCTGGTGTAGC
**ChIP ERdj4**	AAGTGACGCAAGGACCAAAC	GAGTAGCCTCCTCCCAGCTC
**Actin**	GATCTGGCACCACACCTTCT	GGGGTGTTGAAGGTCTCAAA
**Myc-XBP1s-K276R**	GTATTGAGGAAGCACCTCTAAGCT CTTCAGAAG	GCACTACCACGTTAGTTTGACTCTC TGTCTCAG
**Myc-XBP1s-K298R**	GTGAGCCTTTGGAAGATGACTTCA TCCC	GCTTCACTGAGACAATGAATTCAGG GTGATC

Table S3. List of siRNA oligonucleotides

Transcripts	Target sequences
*** si-3'UTR-XBP1**	5'-CAGCUUUUGAGAUUCUAGU-3'<br>5'-CACUGUUGCCUCUUCAGAU-3'<br>5'-CGUGAGACUCGGUCUGGAA-3'
*** si-SHP**	5'-GUCGUCCGACUAUUCUGUA-3'<br>5'-CCCAGUAUACUUAAGAAGA-3'<br>5'-CUCCUGACUUUGUACAGAA-3'
*** si-PIAS1**	5'-CCAUGGCAGUAUAUCUUGU-3'<br>5'-CCUCAACAGUUCUUCUUCA-3'<br>5'-CUCCGAGUAUACUUCCUUU-3'
**si-PIAS2**	5'-AGUCUUCGAGUGUCCUUGA-3'
*** si-PERK**	5'-CCAUACUACAAGAGAGAAA-3'<br>5'-GCGUUGUCUUUGAAGCUAA-3'<br>5'-GAACUCCUCUACCCAUUCA-3'
*** si-IRE1**	5'-GAUAGUCUCUGCCCAUCAA-3'<br>5'-GUAAGUGACCGAAUAGAAA-3'<br>5'-GACUAAGCUUCGCAAAUCA-3'
*** si-ATF6**	5'-CGACUGUGGUUCAACUUCA-3'<br>5'-GACUAAACCUGUUCUACAA-3'<br>5'-CCACAACAAGACCACAAGA-3'

* : A mixture of 3 independent siRNA oligonucleotide.

Table S4. List of primary antibodies used in this work

				Working dilution		
**Primary antibody**	**Supplier (Cat.Number)**	**RRID**	**Host**	**IB**	**IF**	**IP**
Anti-phospho-eIF2α	Cell signaling Technology (3398)	AB_2096481	rabbit	1:1000		
Anti-ATF4	Cell signaling Technology (11815)	AB_2616025	rabbit	1:1000		
Anti-CHOP	Thermo Fisher Scientific (MA1-250)	AB_2292611	mouse	1:1000	1:200	
Anti-BIM	Abcam (ab32158)	AB_725697	rabbit	1:1000		
Anti-caspase 12	Cell signaling Technology (2202)	AB_2069200	rabbit	1:1000		
Anti-cleaved caspase 3	Cell signaling Technology (9664)	AB_2070042	rabbit	1:1000		
Anti-EDEM1	Abcam (ab200645)		rabbit	1:1000		
Anti-HRD1(SYVN1)	Cell signaling Technology (14773)	AB_2798607	rabbit	1:1000		
Anti-BiP	Cell signaling Technology (3177)	AB_2119845	rabbit	1:1000		
Anti-ERdj4(DNAJB9)	Abcam (ab200645)	AB_10899644	goat	1:1000		
Anti-phospho-IRE1α	Abcam (ab48187)	AB_873899	rabbit	1:1000		
Anti-IRE1α	Abcam (ab37073)	AB_775780	rabbit	1:1000		
Anti-XBP1	Santa Cruz Biotechnology (sc-8015)	AB_628449	mouse		1:100	1:100
Anti-XBP1	Santa Cruz Biotechnology (sc-7160)	AB_794171	rabbit	1:1000		
Anti-GAPDH	Santa Cruz Biotechnology (sc-48167)	AB_1563046	goat	1:1000		
Anti-SP1	Santa Cruz Biotechnology (sc-14027)	AB_2171049	rabbit	1:1000		
Anti-PIAS1	Cell signaling Technology (3550)	AB_1904090	rabbit	1:1000		
Anti-PIAS1	Santa Cruz Biotechnology (sc-365127)	AB_10707973	mouse			1:100
Anti-PIAS2	Abcam (ab126601)	AB_11128710	rabbit	1:1000		
Anti-PIAS3	Cell signaling Technology (4164)	AB_1904092	rabbit	1:1000		
Anti-PIAS4	Cell signaling Technology (4392)	AB_10547884	rabbit	1:1000		
Anti-SUMO1	Cell signaling Technology (4930)	AB_10698887	rabbit	1:1000	1:100	
Anti-SHP	Abcam (ab186874)	AB_2797389	rabbit	1:1000		
Anti-phospho-PERK	Thermo Fisher Scientific (MA5-15033)	AB_10980432	rabbit	1:1000		
Anti-PERK	Cell signaling Technology (5683)	AB_10841299	rabbit	1:1000		
Anti-ATF6	Abcam (ab11909)	AB_298691	rabbit	1:1000		
Anti-Ubiquitin	Cell signaling Technology (3933)	AB_2180538	rabbit	1:1000		
Anti-β-actin	Santa Cruz Biotechnology (sc-47778)	AB_2714189	mouse	1:1000		
Anti-Myc	Cell signaling Technology (2278)	AB_490778	rabbit	1:1000		
Anti-GFAP	Cell signaling Technology (3670)	AB_561049	mouse		1:100	
Anti-MAP2	Abcam (ab32454)	AB_776174	rabbit		1:100	
Anti-Tyrosine Hydroxylase	Pel-Freez Biologicals (P40101)	AB_2313713	rabbit		1:500	
